# New Insights into the Development of Papillary Thyroid Cancer: The Roles of miR-1179 and ELF3

**DOI:** 10.3390/cells15090802

**Published:** 2026-04-29

**Authors:** Nicolas Henry, Nisrine Bahassou, Frédérick Libert, Geneviève Dom, Carine Maenhaut

**Affiliations:** 1 IRIBHM J. E. Dumont, Université Libre de Bruxelles, 1070 Brussels, Belgium; 2 BRIGHTcore Facility, 1070 Brussels, Belgium

**Keywords:** miR-1179, miRNA, ELF3, NOTCH3, CX3CL1, papillary thyroid carcinoma, thyroid

## Abstract

Thyroid cancer is the most prevalent endocrine malignancy, and papillary thyroid carcinoma (PTC) is the most common type of thyroid cancer. Although the prognosis is generally favorable, a better understanding of the molecular mechanisms involved in this pathology could lead to new treatment opportunities. Dysregulation of miRNA expression has been correlated with tumor development, and miR-1179 has been previously identified as one of the most downregulated miRNAs in PTC. This study aimed to explore the role of miR-1179 in thyroid tumorigenesis. miR-1179 was overexpressed in the TPC-1, B-CPAP, and HTori-3 thyroid cell lines to characterize its function and identify mRNA targets. The relevance of our data for human PTC was then addressed by analyzing TCGA and independent PTC. We showed that miR-1179 triggered apoptosis and inhibited cell migration. We identified ELF3 as a direct target of miR-1179 and other effectors, including NOTCH3 and CX3CL1. Finally, we revealed the existence of an inverse correlation between decreased expression of miR-1179 and increased expression of *ELF3*, *NOTCH3*, and *CX3CL1* mRNA in human PTC. Our findings suggest that miR-1179 is a tumor suppressor gene and that its loss may contribute to thyroid tumor progression by promoting the expression of ELF3, NOTCH3, and CX3CL1.

## 1. Introduction

Papillary thyroid carcinoma (PTC) is the most common type of thyroid malignancy, accounting for approximately 85% of all thyroid cancers, with an incidence rising consistently over each year. PTC is generally a well-differentiated tumor associated with a favorable prognosis [1,2,3]. The initiation and progression of PTC are strongly driven by genetic alterations in the mitogen-activated protein kinase (MAPK) pathway. Up to 70% of PTCs have somatic mutations in genes coding for this pathway. The main mutations are activating mutations in the *BRAF* (40–50%) and *RAS* genes (10–20%), as well as *RET/PTC* chromosomal rearrangements (10–20%) [4,5]. Despite this favorable prognosis, 5–20% of cases exhibit recurrences, and 10–15% develop distant metastases, progressing to poorly differentiated thyroid carcinoma (PDTC) or anaplastic thyroid carcinoma (ATC), which are associated with worse clinical outcomes [1].

A major challenge in thyroid carcinoma lies in accurate diagnosis. Most patients are asymptomatic, and tumors are often detected incidentally through the identification of intrathyroidal nodules during medical examination. A substantial proportion of these nodules remain indeterminate, often leading to diagnostic thyroidectomy [6]. Although effective, this procedure requires lifelong hormone replacement therapy, underscoring the need for reliable diagnostic biomarkers. In recent years, targeted therapies have been developed for tumors resistant to conventional treatments such as chemotherapy and radiotherapy, as well as for radioiodine-refractory tumors, with a particular focus on the BRAF^V600E^ mutation due to its central role in tumorigenesis [7,8]. Despite these advances, the emergence of drug resistance remains a major clinical problem, highlighting the need for a better understanding of the molecular mechanisms underlying tumorigenesis in order to propose alternative therapies.

MicroRNAs (miRNAs) have emerged as promising biomarkers to distinguish benign from malignant thyroid nodules and for classifying thyroid cancer subtypes [9,10,11,12,13]. miRNAs are a class of small non-coding RNAs, 19 to 25 nucleotides in length, that negatively regulate gene expression at the post-transcriptional level through binding to the 3′ untranslated region (3′UTR) of target mRNAs, and are predicted to regulate the majority of the human transcriptome [14]. Numerous studies have shown that dysregulation of miRNA expression is linked to the onset and progression of tumors and have revealed their potential as valuable tools for the development of innovative therapeutic approaches [15,16,17].

Our previous miRNA sequencing analysis of PTC and associated lymph node metastases has revealed various miRNAs that are up- and downregulated in the tumors, with miR-1179 among the most significantly downregulated [18]. This miRNA is encoded by the *MIR1179* gene, which is located in an intergenic region as a bicistronic *MIR7-2/MIR1179* locus on chromosome 15 (15q26.1), whose upregulation and consequent overexpression of both miRNAs have been correlated with altered thyroid function and thyrotropin resistance [19,20]. Furthermore, genome-wide association studies identified an SNP in a locus near *MIR1179* (rs17776563), which has been linked to variation in TSH levels [21]. Analyses of human cohorts show that miR-1179 expression is deregulated in thyroid tumors but also in various other tumors, where it may play a role as an oncogene or tumor suppressor gene, such as in colorectal, pancreatic, gastric, breast, and lung cancer. Its deregulation impacts different biological processes such as proliferation, apoptosis, migration, and invasion, as demonstrated by in vitro analyses [22,23,24,25,26].

In thyroid tumors, although several groups reported a decreased expression of miR-1179 in PTC [27,28,29], in accordance with our data [18], its functional characterization in the thyroid remains limited. In vitro studies suggest that miR-1179 may promote PTC tumorigenesis or aggressiveness by targeting ABCA9 or RAB23 [30,31]. However, these investigations primarily focus on the role of circRNAs in the thyroid context. The precise role of miR-1179 in normal thyroid function, as well as the functional consequences of its downregulation in thyroid tumor development, have not yet been elucidated.

In this study, we aimed to investigate the biological role of miR-1179 in papillary thyroid tumorigenesis. To this end, we performed a series of functional assays and identified the molecular pathways modulated following miR-1179 expression in different thyroid cell lines. Our results show that this miRNA plays an important role in triggering apoptosis and inhibiting cell migration. We identified the ELF3 transcription factor as a direct target of this miRNA, and other effectors, including NOTCH3 and CX3CL1. We then confirmed the relevance of our results for human PTC by performing in silico analyses of the TCGA database and in vivo investigations of independent PTC samples.

## 2. Materials and Methods

### 2.1. Tissue Collection

Papillary thyroid tumors and adjacent normal thyroid tissues (*n* = 11) were obtained from the J. Bordet Institute. Collected tissues were immediately dissected, embedded in OCT (optimal cutting temperature), and frozen at −80 °C. All samples were stained with hematoxylin and eosin, and their pathological status was confirmed by an anatomopathologist from the J. Bordet Institute. This study was conducted in accordance with the Declaration of Helsinki and approved by the Ethics committee of the J. Bordet Institute (Brussels, Belgium, protocol 1978-01/12/2016). All participants in the study gave written informed consent. Clinical information is summarized in Supplementary Table S1.

### 2.2. In Silico Analysis

Human thyroid samples (460 primary papillary thyroid cancers, 59 normal, and 7 metastatic tissues, including all histological subtypes) data were obtained from The Cancer Genome Atlas (TCGA) via the UCSC Xena Browser (https://xenabrowser.net/, accessed on 1 November 2025). TCGA thyroid cancer (THCA) datasets were analyzed for the expression of hsa-miR-1179, *ELF3*, *NOTCH3*, *CX3CL1*, and *ALPK2*.

### 2.3. Cell Lines and Treatments

The TPC-1 cell line is derived from a PTC with the RET/PTC1 rearrangement and was obtained from Pr. M. Mareel (University of Ghent, Ghent, Belgium) [32]. The B-CPAP cell line is derived from a BRAF^V600E^-positive poorly differentiated thyroid carcinoma (PDTC) and was received from Prof. G. Brabant (Department of Internal Medicine I, Lübeck, Germany). The non-tumorigenic HTori-3 cell line is derived from human normal thyroid epithelial cells immortalized in vitro by transfection with simian virus 40 (SV40) DNA [33]. STR profiling was performed to guarantee their purity and identity. All cell lines were maintained in RPMI 1640 medium (52400025, ThermoFisher Scientific, Dilbeek, Belgium) supplemented with 10% fetal bovine serum (FBS), 2% penicillin/streptomycin, and 1% amphotericin B at 37 °C in a humidified atmosphere containing 5% CO_2_. All cell lines were used below passage 18 and were routinely tested to confirm they were free of mycoplasma contamination.

Cells were treated with demethylating agent (5′-Aza-2-deoxycytidine (5′-Aza), 10 µM) or histone deacetylase inhibitor (suberoylanilide hydroxamic acid (SAHA), 1 µM) for 24 h and then harvested for RNA extraction.

### 2.4. Generation of Stable TPC-1-miR-1179 Inducible Cell Lines

Third-generation lentiviral vectors were produced as previously described by Dull et al. [34]. HEK293T cells were co-transfected with a transfer plasmid expressing an shMIMIC miR-1179 (GSH11926-213609830, Dharmacon Reagents, Lafayette, CO, USA), a packaging plasmid expressing Gag/Pol, and an envelope plasmid encoding VSV-G. Culture supernatants were harvested 48 h post-transfection, clarified by centrifugation to remove cellular debris, filtered, and used to transduce TPC-1 cells. Vector expression was induced with 100 ng/mL of doxycycline. Following clonal selection, individual clones were first screened for the absence of phenotypic alterations compared to the parental TPC-1 cell line, with particular attention to cell viability. Clones meeting this condition were then evaluated for inducibility through tRFP flow cytometry and RT-qPCR quantification of hsa-miR-1179 across a range of doxycycline concentrations. Based on these analyses, two independent inducible TPC-1-miR-1179 (TPC-1-1179i) cell lines, designated 2G9 and 1D5, were selected for their stable phenotype under non-induced conditions and their robust and consistent miR-1179 induction upon doxycycline treatment, and were subsequently used for all further experiments.

### 2.5. Cell Transient Transfection

Cells were transiently transfected with the miRIDIAN microRNA mimic hsa-miR-1179 (C-301320-00-0005, Horizon Discovery, Cambridge, UK) and a negative control (Cel-miR-67, NC-001000-01-05, Horizon Discovery) at a final concentration of 5 nM, or with siRNA Silencer Select targeting ELF3 (SI04265660, Qiagen, Antwerpen, Belgium) and a corresponding negative control (4390846, ThermoFisher Scientific) at a final concentration of 20 nM. Transfections were performed using Lipofectamine RNAiMAX reagent (13778150, ThermoFisher Scientific) according to the manufacturer’s instructions. Overexpression of miR-1179 and silencing of ELF3 were verified after each transfection by RT-qPCR analysis.

### 2.6. RNA Purification and Real-Time PCR Analysis

The total RNA fraction was extracted from cells using the miRNeasy Mini Kit (217004, Qiagen) according to the manufacturer’s instructions. RNA purity was evaluated using the NanoDrop Eight Spectrophotometer (ThermoFisher Scientific) by measuring the absorbance ratio at 260/280 and 260/230 nm. Superscript III reverse transcription kit (18080093, Thermofisher Scientific) and miRCURY LNA RT kit (339340, Qiagen) were used to reverse transcribe the mRNA and miRNA fractions, respectively. PCR amplification was performed using KAPA SYBR FAST (KK4601, KapaBiosystems, Wilmington, MA, USA) and the miRCURY LNA SYBR Green PCR kit (339345, Qiagen) to respectively quantify mRNA and miRNA, using the ABI 7500 detection system (Biorad, Temse, Belgium). Two internal normalizers were used for mRNA quantification: *NEDD8*, *TTC1* [35], and one for miRNA quantification: U6 snRNA. The relative gene expression was calculated and normalized according to the 2^−∆∆Ct^ method [36]. Primers for miR-1179 and U6 snRNA used for miRNA quantification were purchased from Qiagen (YP00204624; YP00203907). Primer sequences used for mRNA quantifications are listed in Supplementary Table S2.

### 2.7. RNA Sequencing Analysis

RNA sequencing was conducted at the BRIGHTcore facility (ULB/VUB, UZ Brussels, 1090 Brussels, Belgium). RNA integrity was assessed using a Fragment Analyzer (Agilent Technologies, Santa Clara, CA, USA), and only samples with high-quality RNA (RQN ≥ 7.5) were used. Stranded cDNA libraries were generated with the TruSeq Stranded mRNA Sample Prep Kit (20020595, Illumina, San Diego, CA, USA) according to the manufacturer’s protocol. Libraries were multiplexed and sequenced on a NovaSeq 6000 platform (Illumina) using a 200-cycle kit. Sequencing depth per sample, mapping rate, and alignment statistics were monitored for quality control; no samples were excluded based on QC metrics. Paired-end reads were aligned to the GRCh38 human reference genome using STAR v2.7.0f, with annotations from Ensembl (Homo_sapiens.GRCh38.90.gtf). Gene-level counts were obtained after transcript assembly with HTSeq v0.9.1 and normalized to 20 million aligned reads. Each experimental condition was performed in three independent biological replicates. Differential expression between experimental conditions was determined using iDEP 2.0, with the EdgeR quasi-likelihood method applied for statistical testing. RNA-seq candidates were selected using FDR < 0.1 and fold-change > 1.5, reflecting an exploratory analysis.

### 2.8. Cell Counting

Cells were seeded in 6-well plates, and cell numbers were assessed between days 1 and 4 using a Neubauer chamber. On day 0, 1.5 × 10^5^ cells were seeded for the TPC-1-miR-1179 inducible (TPC-1-1179i) cell lines, and 2.5 × 10^6^ cells were seeded for the transiently transfected cells.

### 2.9. Cell Viability Assay (MTS)

Cells (5 × 10^3^) were seeded in 12-well plates, and cell viability was assessed between days 4 and 6 using the CellTiter 96^®^ AQueous Non-Radioactive Cell Proliferation Assay (G5421, Promega, Leiden, The Netherlands), according to the manufacturer’s instructions. Following incubation with the MTS reagent, absorbance of the soluble formazan product was measured at 490 nm using a microplate reader (Bio-Rad, San Francisco, CA, USA). All values correspond to the raw absorbance measured each day, after subtracting the blank value (medium alone).

### 2.10. Cell Migration and Invasion Assay

Cell migration and invasion were assessed using 24-well Transwell chambers with 8 µm pore size, according to the manufacturer’s instructions. For invasion assays, chambers were coated with Matrigel (VWR-353097 and VWR-354480, Corning, Leuven, Belgium). Cells were harvested 72 h after induction or transient transfection, and 4 × 10^4^ cells were seeded into the upper chambers in serum-free medium. The lower chambers contained complete medium to serve as a chemoattractant. 20 h after incubation, migrative and invasive cells were fixed and stained using Diff-Quik (26419, Polysciences, Taipei, Taiwan). Migrative and invasive cells were quantified by counting cells in five randomly selected fields and acquired using a Bio-Rad ZOE Fluorescent Cell Imager.

### 2.11. Apoptosis Assay

Apoptosis was assessed using different complementary approaches, and staurosporine-treated cells (10 µM, S5921, Merck Life Science, Hoeilaart, Belgium) were used as a positive control. Phosphatidylserine externalization was measured by Annexin V staining (V13242, ThermoFischer Scientific) four days after induction or 24 h after transfection, according to the manufacturer’s instructions, and analyzed by flow cytometry. Caspase-3/7 activity was quantified using the Caspase-Glo^®^ 3/7 assay (G8090, Promega). For this assay, 5 × 10^3^ cells were seeded in 96-well plates 3 days after transfection to normalize cell density. 20 h after cell seeding, the Caspase-Glo^®^ 3/7 reagent was added, and the cells were incubated for 60 min. The supernatant was transferred to a white 96-well plate, and luminescence was measured using the Infinite 200 Pro luminometer (TECAN, Mechelen, Belgium). Blank control values were subtracted from all readings, and each data point represents the mean of two technical replicates.

### 2.12. Proliferation Assay

Cell proliferation was assessed by EdU (5-ethynyl-2′-deoxyuridine) incorporation using the Click-iT™ EdU Flow Cytometry Kit (C10633, ThermoFisher Scientific). Three days following induction or transient transfection, cells were incubated with 10 µM EdU for 3 h, according to the manufacturer’s instructions, and EdU incorporation was quantified by flow cytometry.

### 2.13. Flow Cytometry Analysis

Flow cytometry analyses were conducted on a BD LSR Fortessa (Becton Dickinson, Erembodegem, Belgium) equipped with four lasers (violet, blue, yellow/green, and red). All data were acquired and analyzed using BD FACS Diva software (version 9). Compensation and doublet discrimination were applied for all experiments, and a minimum of 10,000 events per sample was recorded.

### 2.14. Western Blotting

Cells were washed twice with dPBS (Dulbecco’s Phosphate-Buffered Saline), and proteins were extracted by lysis on ice, using Laemmli buffer supplemented with protease and phosphatase inhibitors. Protein concentrations were determined by spectrophotometry at 280 nm using the NanoDrop Eight Spectrophotometer (ThermoFisher Scientific). For each experiment, 30–40 µg of denatured protein were resolved on an 8% polyacrylamide gel and transferred to nitrocellulose membranes. Membranes were incubated overnight at 4 °C with primary antibodies against ELF3 (AB133621, Abcam, Cambridge, UK, 1/1000), NOTCH3 (AB23426, Abcam, 1/1000), vinculin (VIN-54, ab130007, Abcam, 1/1000) or actin (ACTA1, A2066, Merck, 1/1000), followed by HRP-conjugated secondary antibodies (Peroxidase AffiniPure Donkey Anti-Mouse IgG (H+L) (715-035-150, Jackson ImmunoResearch, West Grove, PA, USA, 1/3750) or Peroxidase AffiniPure Donkey Anti-Rabbit IgG (H+L) (711-035-152, Jackson ImmunoResearch, 1/3750)) for 1 h at room temperature. Protein signals were detected using the Western Lightning Plus-enhanced chemiluminescence substrate (NEL103E001EA, Perkin Elmer, Waltham, MA, USA) and normalized to actin or vinculin. Signal intensities were quantified using ImageJ software (version 1.54d).

### 2.15. Luciferase Reporter Assay

A fragment of the human *ELF3* mRNA 3′ untranslated region (3′UTR) containing the predicted miR-1179 binding site, either in its wild-type form or carrying mutations within the predicted miR-1179 target sequence, was inserted downstream of the Renilla luciferase gene coding sequence in the psiCHECK-2 reporter plasmid (C8021, Promega). No additional potential miR-1179 binding sites are present within the cloned fragment. TPC-1 and HTori-3 cells were co-transfected with 1 µg of the reporter construct and 50 nM of either hsa-miR-1179 mimic or negative control using Lipofectamine 3000 (L3000015, ThermoFisher Scientific) following the manufacturer’s instructions. Twenty-four hours after transfection, Renilla and Firefly luciferase activities were quantified with the Dual-Glo^®^ Luciferase Assay System (E2920, Promega). Each experiment was performed in at least three independent biological replicates, and for each replicate, measurements were obtained in technical duplicates. Raw luminescence values were corrected by subtracting blank measurements, and the ratio of Renilla to Firefly luminescence was calculated to normalize transfection efficiency and determine relative luciferase activity.

### 2.16. Statistical Analyses

Statistical analyses were performed using GraphPad Prism version 8.4.3 (GraphPad Software, San Diego, CA, USA). Normality was assessed with the Shapiro–Wilk test. Paired tests were applied when measurements were obtained from matched or repeated samples. Specifically, for normally distributed data, RM one-way ANOVA with Geisser–Greenhouse correction (for ≥3 conditions) and paired *t*-tests (for 2 conditions) were used. When normality was not met, the Friedman test (for ≥3 conditions) and the Wilcoxon signed-rank test (for 2 conditions) were applied. Unpaired tests were applied for independent groups, and the Kruskal–Wallis test with Dunn’s post hoc analysis (for ≥3 groups) was used since normality was not met.

Independent biological replicates were defined as separately cultured cells or independently processed patient samples; repeated measurements on the same preparation were treated as technical replicates. Results are presented as median ± interquartile range (IQR) from ≥3 independent biological replicates (*n* = independent experiments), with *p* < 0.05 considered significant.

## 3. Results

### 3.1. miR-1179 Expression Is Downregulated in Thyroid Carcinomas and Thyroid-Derived Cell Lines

**Figure 1:**
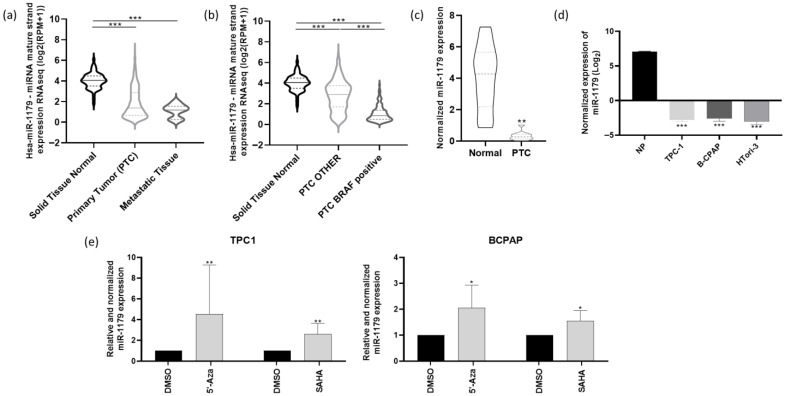
miR-1179 is downregulated in human papillary thyroid cancer and thyroid-derived cell lines, and its expression can be restored by a demethylation agent or a histone deacetylase inhibitor. (**a**–**c**) miR-seq analysis of miR-1179 expression levels from The Cancer Genome Atlas (TCGA). (**a**) In normal thyroid samples (*n* = 59), PTC (*n* = 460), and metastatic tissues (*n* = 7). (**b**) In normal thyroid samples (*n* = 59), PTC harboring the BRAF^V600E^ mutation (PTC BRAF-positive, *n* = 250) and PTC with other genetic alterations (PTC OTHER, *n* = 210). (**c**,**d**) miR-1179 expression level analysis by RT-qPCR (**c**) in independent PTC and adjacent normal thyroid tissues (*n* = 11), and (**d**) in the TPC-1, B-CPAP, and HTori-3 cell lines (*n* = 4) and a pool of 8 normal human thyroid tissues (NP). (**e**) miR-1179 expression level analysis by RT-qPCR in TPC-1 and B-CPAP treated for 24 h with DMSO, 5′-aza-2-deoxycytidine (5′-Aza, 10 µM), and suberoylanilide hydroxamic acid (SAHA, 1 µM). Data analyzed with the Kruskal–Wallis test, except (**c**,**e**), for which the Wilcoxon signed-rank test (* *p* < 0.05, ** *p* < 0.01, *** *p* < 0.001) was used.

We first analyzed the miR-seq data from The Cancer Genome Atlas (TCGA), focusing on the THCA (Thyroid cancer) cohort, which includes 59 normal thyroid tissues, 460 primary papillary thyroid cancer (PTC) samples, and seven metastatic samples. The expression of miR-1179 was significantly downregulated in both primary tumors and metastatic samples compared to normal tissues (Figure 1a). THCA data further revealed that miR-1179 expression was significantly lower in BRAF^V600E^-positive PTC compared to PTC harboring other genetic alterations (Figure 1b). The downregulation of miR-1179 was confirmed by RT-qPCR in eleven independent PTC samples compared with their associated non-tumoral adjacent tissues (Figure 1c, clinicopathological data in Supplementary Table S1), supporting the data previously obtained in our laboratory [18]. We next analyzed miR-1179 expression in three thyroid-derived cell lines. We selected one classical PTC-derived cell line harboring the *RET/PTC1* rearrangement, one of the most frequent *RET* rearrangements in PTC (TPC-1), and one BRAF^V600E^-positive cell line (B-CPAP). Both cell lines cover the main oncogenic alterations present in PTC. To our knowledge, no *RAS*-mutated PTC-derived cell line currently exists. In addition to those two cancer-derived cell lines, the non-tumorigenic HTori-3 cell line, derived from SV40-immortalized normal thyrocytes, was included to investigate the role of miR-1179 in a non-tumoral context. RT-qPCR analysis revealed a significant decrease in miR-1179 expression in all three cell lines compared to normal human thyroid tissues (Figure 1d). We further investigated the mechanisms underlying the downregulation of miR-1179 expression. Given that epigenetic silencing events are known to occur during tumorigenesis, we examined whether the miR-1179 promoter might be hypermethylated or whether its accessibility could be restricted due to alterations in the acetylation of nearby histones. To address this, we treated TPC-1 and B-CPAP cells for 24 h with 5′-aza-2′-deoxycytidine (5′-Aza, 10 µM), a demethylating agent, or with suberoylanilide hydroxamic acid (SAHA, 1 µM), a histone deacetylase inhibitor. RT-qPCR analyses revealed that both cell lines exhibited re-expression of miR-1179 following the treatments (Figure 1e).

### 3.2. miR-1179 Overexpression Inhibits Cell Growth and Migration, Induces Apoptosis, but Does Not Affect Proliferation or Invasion

To investigate the biological function of miR-1179, we performed gain-of-function experiments by generating stable TPC-1 cell lines with doxycycline-inducible miR-1179 expression. These doxycycline-inducible TPC-1-derived cell lines (TPC-1-1179i) are referred to as 2G9 and 1D5. Upon vector induction (TPC-1-1179i+: 2G9+, 1D5+), a bicistronic construct encoding the *miR-1179* shRNA and the fluorescent protein TurboRFP (tRFP) is transcribed. We therefore first verified vector induction following doxycycline addition by assessing tRFP expression using flow cytometry, revealing an increase in tRFP-positive cells (Supplementary Figure S1A). We then measured miR-1179 expression levels by RT-qPCR and showed that these levels were increased, confirming vector induction (Supplementary Figure S1B). However, these RT-qPCR analyses also revealed that, in the absence of doxycycline, the TPC-1-1179i cell lines already exhibited a low but significant basal upregulation of miR-1179 compared to the wild-type (WT) TPC-1 cells, suggesting a leak of the expression cassette. Although this prevented us from having the corresponding controls (uninduced TPC-1-1179 cells), we saw this as an opportunity to analyze the impact of a moderate increase in miR-1179 levels compared to the more important increase observed following doxycycline induction. Therefore, in our experiments, we included WT TPC-1 cells as control cells (low levels of miR-1179), non-induced TPC-1-1179i cells (moderate levels of miR-1179 resulting from the leak of the expression cassette), and doxycycline-induced TPC-1-1179i cells (high levels of miR-1179). No difference in miR-1179 expression was observed between WT TPC-1 cells treated or not with doxycycline (Supplementary Figure S2A). To further validate the results obtained with our inducible models, we also performed transient transfections of TPC-1, B-CPAP, and HTori-3 cells using a hsa-miR-1179 mimic (miR-1179) or a negative control mimic (miR-NC: Cel-miR-67). The transfection efficiencies were assessed by FACS and fluorescence microscopy using the fluorescent mimic SIGLO and were approximately 95% for the three cell lines, as previously shown in our previous studies [37,38]. In the three cell lines, miR-1179 expression was analyzed at days 1, 3, 4, and 7 post-transfection, and was significantly increased compared to the negative control condition (Supplementary Figure S1C). For clarity, we will present in the main manuscript the results obtained with the inducible expression of miR-1179 (TPC-1-1179i cell lines: 2G9 and 1D5), all the results related to the miR-1179 transient transfection experiments in the TPC-1, B-CPAP, and HTori-3 cell lines being provided as Supplementary Data. In addition, data relating to wild-type TPC-1 cells treated with doxycycline (TPC-1+ cells) are also included there.

**Figure 2:**
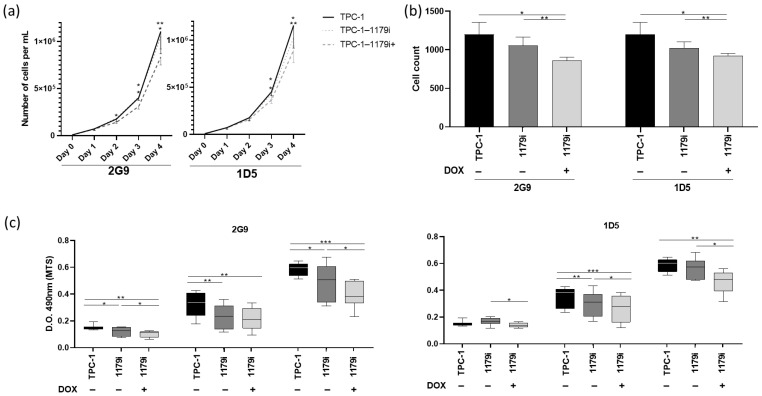
miR-1179 induction inhibits cell growth in the TPC-1-1179i cell lines (2G9 and 1D5). Cells were treated with 100 ng/mL doxycycline (TPC-1-1179i+ cells). (**a**) Growth kinetics assay: total cell number was quantified daily for each condition by manual counting using a Neubauer chamber (*n* = 5) (two-way ANOVA, * *p* < 0.05, ** *p* < 0.01) (**b**) Cell counts at day 3 (RM one-way ANOVA, * *p* < 0.05, ** *p* < 0.01). (**c**) Cell viability analysis by absorbance measurement at 490 nm (MTS test) and reported as mean values over time for each condition (*n* = 8) (Friedman test, * *p* < 0.05, ** *p* < 0.01, *** *p* < 0.001).

We first assessed cell population growth by performing cell counting experiments. Kinetic analyses from day 0 to day 4 in the TPC-1-1179i cell lines revealed a significant reduction in cell numbers three and four days after doxycycline addition in the TPC-1-1179i+ cells compared to both untreated TPC-1-1179i and WT TPC-1 cells (Figure 2a,b). Similar results were obtained after transient transfection of miR-1179 in the TPC-1, B-CPAP, and HTori-3 cell lines, with a decrease in cell number observed at day 3 post-transfection in the transfected cells compared to both untransfected (NT) and miR-NC control conditions (Supplementary Figure S3A). In parallel, we also assessed cell viability using an MTS assay. Measurement of optical densities from days 4 to 6 in the two inducible cell lines revealed a decrease in cell viability after miR-1179 induction at days 4, 5, and 6 (Figure 2c). This decrease in cell viability was also observed, but to a lesser extent, in the non-induced TPC-1-1179i cells at some time points (e.g., 2G9 day 4, 1D5 day 5), reflecting the leak of the expression cassette (Figure 2c).

**Figure 3:**
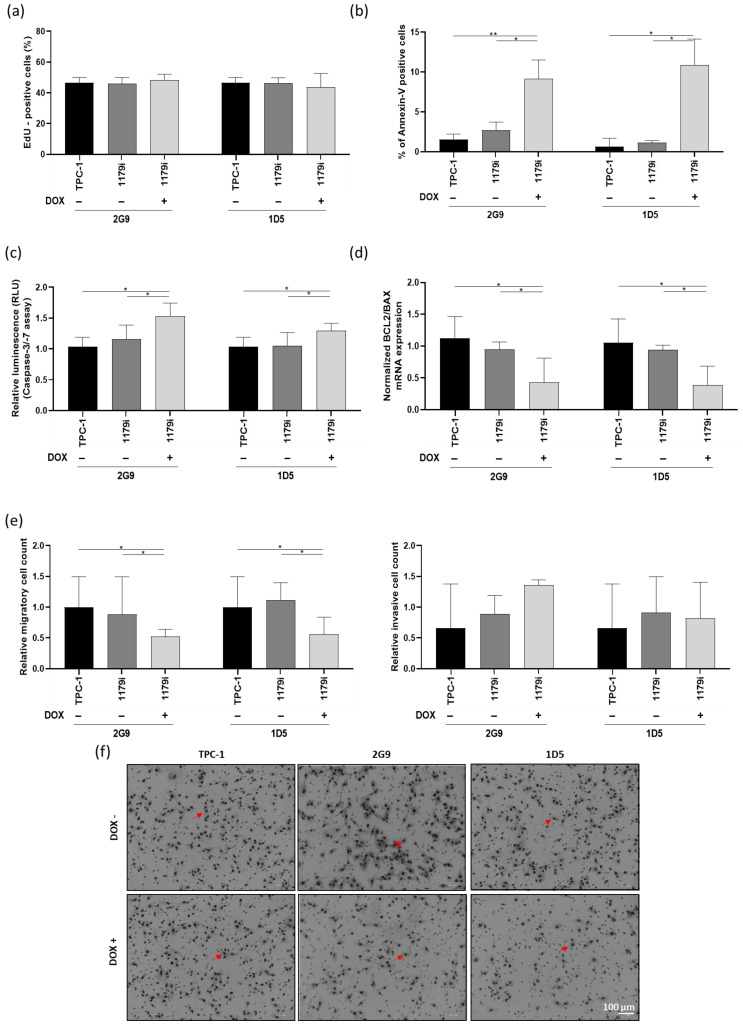
miR-1179 induction does not affect proliferation or invasion but increases apoptosis and reduces migration in the TPC-1-1179i+ cell lines (2G9+ and 1D5+). Cells were treated with 100 ng/mL doxycycline, and the assays were performed 4 days later. (**a**) Cell proliferation assessed by EdU incorporation and quantified by flow cytometry (*n* = 4). Apoptosis assessed by (**b**) Annexin V staining using flow cytometry (2G9, *n* = 7; 1D5, *n* = 6), (**c**) Caspase-3 and -7 activation measured by luminescence (RLU) assays, with values normalized to background levels and relativized to TPC-1 cells treated with doxycycline 100 ng/mL (*n* = 9) and (**d**) *BCL2* and *BAX* mRNA expression analyzed by RT-qPCR, and expressed as the *BCL2*/*BAX* mRNA expression ratio (*n* = 4). (**e**) Quantification of migratory and invasive cell numbers from five random fields per condition, with values relativized to TPC-1 cells treated with doxycycline 100 ng/mL (Transwell assay, *n* = 6). (**f**) Representative images of the migration assay. The red arrows point to examples of cells. All data were analyzed using RM one-way ANOVA, except (**b**), for which the Friedman test was used, * *p* < 0.05, ** *p* < 0.01.

To determine the origin of the decrease in cell number over time following miR-1179 overexpression, we performed an EdU assay at day 3, which indicated that there was no difference in EdU incorporation into DNA during the S phase (Figure 3a). The same results were observed following miR-1179 transfection in the different cell lines (Supplementary Figure S3B). We then considered increased cell death to be a potential cause for the decrease. Annexin V staining detected a significantly higher percentage of phosphatidylserine externalization in the cells, indicating increased apoptosis, 4 days after miR-1179 induction, in the two TPC-1-1179i+ cell lines (2G9+ and 1D5+) compared to both TPC-1-1179i and WT TPC-1 cells (Figure 3b). To further validate our findings on apoptosis, we performed additional assays: first, we analyzed caspase-3 and -7 activities at day 4, which showed a significant increase upon miR-1179 induction (Figure 3c). Second, we measured the BCL2/BAX mRNA expression ratio, a key indicator of pro- versus anti-apoptotic signaling, and found that this ratio was decreased by miR-1179, further supporting its role in promoting apoptosis (Figure 3d). Similarly, in the transient transfection experiments, miR-1179-overexpressing cells exhibited a significantly higher percentage of Annexin V-positive cells, a higher level of caspase-3 and -7 activities, and a decreased *BCL2*/*BAX* mRNA expression ratio, compared to non-transfected and miR-NC-transfected cells (Supplementary Figure S3C–E). As alterations in cell motility are also key features of tumor progression, we finally assessed the migratory and invasive capacities of the cells following miR-1179 overexpression. In the TPC-1-1179i+ cells, miR-1179 induction led to a significant reduction in cell migration compared with controls, whereas no significant differences were observed in invasion in the tested conditions (Figure 3e,f). These findings were further confirmed by transiently expressing miR-1179 in TPC-1, B-CPAP, and HTori-3 cells (Supplementary Figure S3E,F).

### 3.3. RNAseq Identifies Several miR-1179-Regulated mRNAs

**Figure 4:**
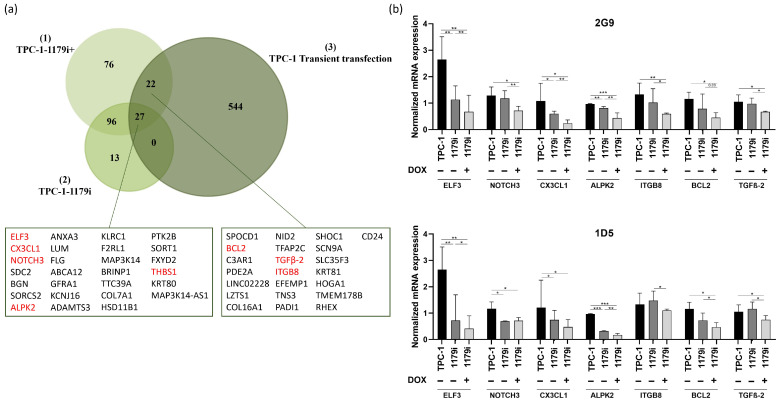
RNA-seq analysis using iDEP 2.0 reveals downregulated mRNAs following miR-1179 expression. Analyses were performed in TPC-1-1179i (2G9 and 1D5) cells treated or not with 100 ng/mL doxycycline (TPC-1-1179i+) and in miR-1179-transfected TPC-1 cells, 4 days after induction or transfection. (**a**) Venn diagram showing the number of differentially expressed genes (DEGs) identified across three iDEP 2.0 analyses: (1) TPC-1-1179i+ vs. TPC-1 cells, (2) TPC-1-1179i vs. TPC-1 cells, and (3) TPC-1 cells transfected with miR-1179 vs. with miR-negative control. Genes selected for further mRNA analyses are indicated in red. (**b**) mRNA expression levels of *ELF3*, *NOTCH3*, *CX3CL1*, *ALPK2*, *ITGB8*, *BCL2*, and *TGFβ-2* quantified by RT-qPCR in the 2G9 and 1D5 cell lines after miR-1179 induction. Sample sizes: 2G9: *ELF3* (*n* = 6), *NOTCH3* (*n* = 6), *CX3CL1* (*n* = 6), *ALPK2* (*n* = 6), *ITGB8* (*n* = 7), *BCL2* (*n* = 5), *TGFβ-2* (*n* = 7); 1D5: *ELF3* (*n* = 6), *NOTCH3* (*n* = 4), *CX3CL1* (*n* = 4), *ALPK2* (*n* = 6), *ITGB8* (*n* = 7), *BCL2* (*n* = 6), *TGFβ-2* (*n* = 7) (RM one-way ANOVA, * *p* < 0.05, ** *p* < 0.01, *** *p* < 0.001).

To further explore the molecular mechanisms underlying the effects of miR-1179 in thyroid cancer cells, we performed RNA sequencing (RNA-seq) on TPC-1-1179i cells, on TPC-1-1179i cells after miR-1179 induction (TPC-1-1179i+), and on TPC-1 cells transiently transfected with an miR-1179 mimic, all harvested four days after induction or transfection. These datasets were compared to their respective control conditions. The analyses were conducted using the iDEP 2.0 platform, and genes displaying at least a 1.5-fold change in expression with a false discovery rate (FDR) < 0.1 were considered differentially expressed. Conserved genes between both models were considered as miR-1179-responsive targets. Given the small increased expression of miR-1179 observed in the non-induced TPC-1-1179i cell lines, this group was also included in the analysis to identify genes modulated by low/moderate miR-1179 expression (Figure 4a). This transcriptomic approach revealed 27 genes that were consistently downregulated across the three experimental conditions, and 22 additional genes that were shared between the transiently transfected cells and the TPC-1-1179i+ cells (Figure 4a).

Among these 49 candidate genes, we selected a few of them for validation and designed specific primers to confirm the gene expression changes identified by transcriptomic analysis using RT-qPCR. These genes were selected for their potential involvement in key tumorigenic processes: *ELF3* (E74 Like ETS Transcription Factor 3), *NOTCH3* (Notch Receptor 3), *CX3CL1* (C-X3-C Motif Chemokine Ligand 1), *ALPK2* (Alpha Kinase 2), *THBS1* (Thrombospondin 1), *ITGB8* (Integrin Subunit Beta 8), *BCL2* (B-Cell Lymphoma 2), and *TGFβ-2* (Transforming Growth Factor Beta 2). Except for *THBS1*, the mRNA downregulation of the others by miR-1179 was validated in the TPC-1-1179i+ cell lines (Figure 4b) and in the transfected TPC-1, B-CPAP, and HTori-3 cell lines, confirming the observations from the RNA-seq analysis (Supplementary Figure S4). Surprisingly, the miR-negative control (miR-NC: Cel-miR-67) appeared to impact the expression levels of most of the investigated mRNAs, essentially in the TPC-1 cells, where they were increased following Cel-miR-67 transfection, and in the B-CPAP cells, where they were decreased compared to non-transfected cells. To account for this variability, we included an additional negative control: cells transfected with miR-204-5p, which, according to existing data, does not target these mRNAs, with the exception of *BCL2* [39]. In all three cell lines, the expression of our selected mRNAs was reduced by miR-1179, whereas it was generally unaffected by miR-204-5p compared to non-transfected cells, confirming the specificity of the action of miR-1179 (Supplementary Figure S4). As expected, *BCL2* mRNA levels were reduced by miR-204-5p in TPC-1 and HTori-3 cells.

### 3.4. ELF3 Is a Direct Target of miR-1179

**Figure 5:**
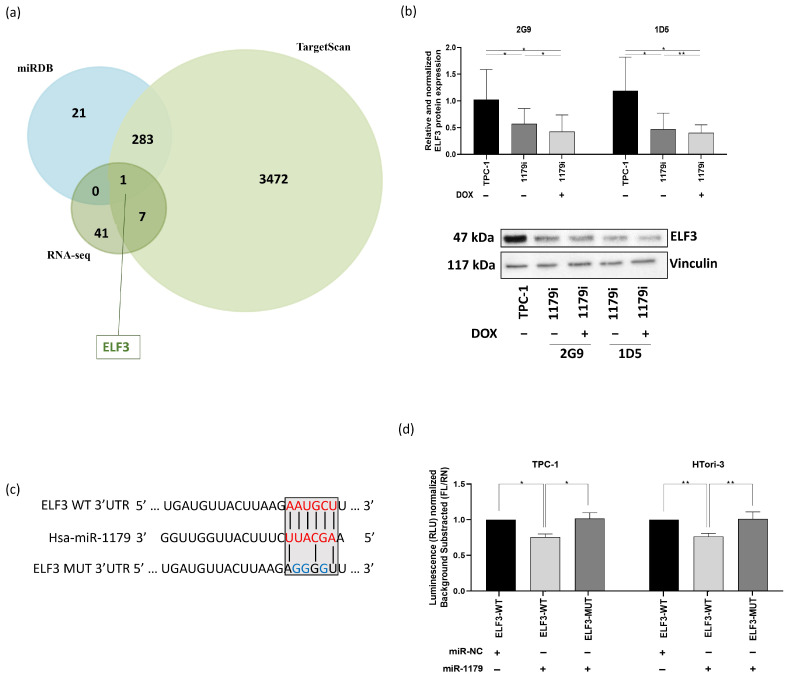
ELF3 is a direct target of miR-1179. (**a**) Venn diagram showing predicted targets of miR-1179 obtained from two online databases (miRDB and TargetScan) and our RNA-seq data. The intersection represents potential direct targets that overlap with the DEGs identified in our RNA-seq analyses. (**b**) ELF3 protein expression assessed by Western blot analysis in the TPC-1-1179i cell lines treated with 100 ng/mL doxycycline (2G9+, 1D5+), normalized to vinculin and relativized to TPC-1 cells treated with 100 ng/mL doxycycline (2G9, *n* = 6; 1D5, *n* = 4). One representative blot is shown. (RM one-way ANOVA, * *p* < 0.05, ** *p* < 0.01). (**c**) Predicted miR-1179 binding site within the *ELF3* 3′UTR (ELF3 WT) and corresponding mutated sequence (ELF3 MUT). The predicted seed-matching nucleotides are highlighted in red, and the mutated residues in blue. (**d**) Luciferase activity (RLU) in TPC-1 and HTori-3 cells co-transfected with ELF3-WT or ELF3-MUT constructs together with either an miR-1179 mimic (ELF3-WT-1179, ELF3-MUT-1179) or negative control mimic (ELF3-WT-NC), with values relativized to ELF3-WT-NC (*n* = 6–8) (Friedman test, * *p* < 0.05, ** *p* < 0.01).

In order to select potential direct targets of miR-1179, we crossed our list of 49 mRNAs with two miRNA target prediction databases, miRDB and TargetScan. From this analysis, only one predicted direct target emerged: *ELF3* (Figure 5a). *ELF3* (E74-like transcription factor 3, *ESE-1*, *ESX*) belongs to the epithelium-specific ETS (ESE) subfamily of ETS transcription factors, which are expressed mainly in epithelial-rich tissues. It plays a key role in regulating epithelial identity and cell fate programs. Dysregulation of *ELF3* expression was shown to be involved in cancer progression, notably through its influence on cell adhesion, proliferation, apoptosis, and EMT-related pathways [40]. *ELF3* thus appeared to be an interesting candidate that could mediate some of the pro-tumor effects resulting from the downregulation of miR-1179.

First, to verify that the decrease in *ELF3* mRNA expression in response to miR-1179 expression did indeed lead to a decrease in the corresponding protein levels, Western blot experiments were performed in the TPC-1-1179i cell lines. Our results showed a significant decrease in ELF3 protein expression after miR-1179 induction (Figure 5b), already present in the absence of doxycycline treatment and displaying an expression gradient parallel to the previously observed *ELF3* mRNA expression gradient (Figure 4b). ELF3 protein expression showed a similar decrease following transient miR-1179 transfection in the three thyroid cell lines and did not change in response to miR-204-5p overexpression included as an additional negative control (Supplementary Figure S5).

To verify whether miR-1179 directly binds to the predicted target site within the 3′UTR region of *ELF3* mRNA, we performed a luciferase reporter assay. A fragment of the *ELF3* 3′UTR, spanning nucleotides 2795–3050 and containing the predicted miR-1179 binding site, was cloned downstream of a constitutively active luciferase reporter and transfected into TPC-1 and HTori-3 cells. The wild-type sequence of the predicted miR-1179 binding site within the *ELF3* 3′UTR, as well as the mutated sequence used to disrupt this interaction, is shown in Figure 5c. Luciferase activity was reduced in cells co-transfected with the wild-type *ELF3* 3′UTR construct and the miR-1179 mimic (ELF3-WT-1179), compared with cells transfected with the negative control mimic (ELF3-WT-NC), suggesting an interaction between miR-1179 and the predicted target site present in *ELF3* (Figure 5d). In contrast, no significant change in luciferase activity was observed in cells transfected with the mutated *ELF3* 3′UTR construct (ELF3-MUT-1179) upon co-transfection with the miR-1179 mimic, validating that miR-1179 is able to specifically and directly bind to the 3′UTR of *ELF3* mRNA.

### 3.5. ELF3 Silencing Reduces Cell Growth, Proliferation, and Migration, and Induces Apoptosis, but Does Not Affect Invasion

**Figure 6:**
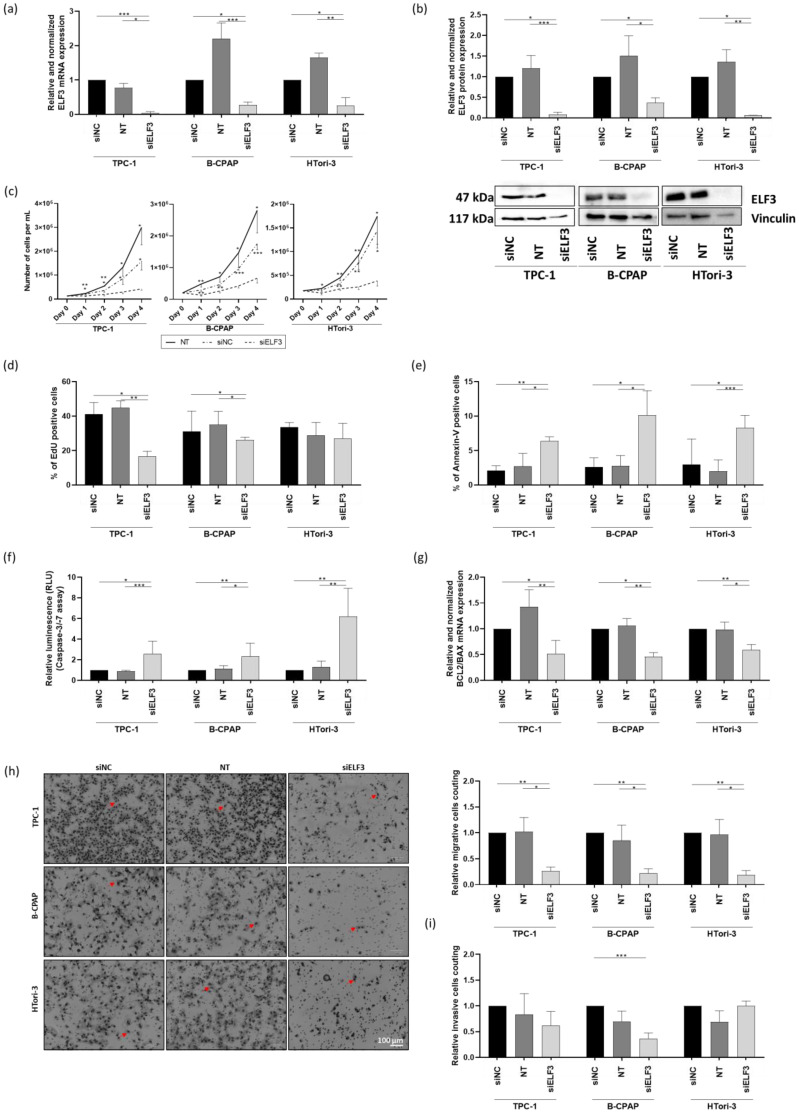
ELF3 knockdown decreases cell growth and EdU incorporation, promotes apoptosis, and inhibits migration in TPC-1, B-CPAP, and HTori-3 cells. (siNC: siRNA-negative control transfected cells, NT: non-transfected cells, siELF3: ELF3 siRNA transfected cells) (**a**) *ELF3* mRNA levels quantified by RT-qPCR four days following siELF3 transfection and relativized to siNC condition (TPC-1, *n* = 8; B-CPAP, *n* = 7; HTori-3, *n* = 10). (**b**) ELF3 protein expression measured by Western blotting four days following siELF3 transfection, normalized to vinculin and relativized to the siNC condition. One representative blot is shown (TPC-1, *n* = 8; B-CPAP, *n* = 4; HTori-3, *n* = 7). (**c**) Time-course analysis of cell number from days 0 to 4, determined daily by Neubauer counting (*n* = 4) (two-way ANOVA, * *p* < 0.05, ** *p* < 0.01, *** *p* < 0.001). (**d**) EdU incorporation analyzed by flow cytometry three days post-transfection (TPC-1, *n* = 6; B-CPAP, *n* = 6; HTori-3, *n* = 4). (**e**) Apoptosis assessed 24 h following siELF3 transfection by Annexin V staining using flow cytometry (TPC-1, *n* = 7; B-CPAP, *n* = 7; HTori-3, *n* = 9) and (**f**) four days post-transfection by Caspase-3/-7 activity measurement by luminescence (RLU) assays, normalized to background and relativized to siNC condition (*n* = 9). (**g**) *BCL2* and *BAX* mRNA levels quantified by RT-qPCR, four days post-transfection, expressed as the *BCL2/BAX* mRNA expression ratio and relativized to the siNC condition (*n* = 7). (**h**) Representative images of the migration assay and quantification of migratory cells from five random fields per condition, four days post-transfection, and relativized to the siNC condition (Transwell assay). The red arrows point to examples of cells (TPC-1, *n* = 7; B-CPAP, *n* = 9; HTori-3, *n* = 9). (**i**) Quantification of invasive cells from five random fields per condition, relativized to the siNC condition (Transwell assay) (*n* = 6). All data, except (**c**), were analyzed using the Friedman test, * *p* < 0.05, ** *p* < 0.01, *** *p* < 0.001.

Very little data exists on the regulation and function of ELF3 in thyrocytes. Therefore, to investigate its functional role and to determine whether its downregulation could reproduce the effects observed upon miR-1179 overexpression, we performed knockdown experiments by transfecting siRNA specifically targeting *ELF3* in the TPC-1, B-CPAP, and HTori-3 cells. We first confirmed by RT-qPCR and Western blotting that siELF3 efficiently reduced both *ELF3* mRNA and protein levels compared to non-transfected cells (NT) and to cells transfected with an siRNA-negative control (siNC) (Figure 6a,b; Supplementary Figure S6).

In the three cell lines, ELF3 knockdown resulted in an important reduction in cell number among the population starting from day 1 to day 4, compared to both siNC-transfected and non-transfected cells (Figure 6c). EdU incorporation into DNA in siELF3-transfected cells decreased in TPC-1 and B-CPAP cells, while no effect was observed in HTori-3 cells (Figure 6d). siELF3-transfected cells showed enhanced apoptosis, as indicated by an increase in the number of Annexin V-positive cells (Figure 6e). This increase in apoptosis was further confirmed by an increase in caspase-3/-7 activities and a decrease in the *BCL2/BAX* mRNA expression ratio (Figure 6f,g). Migration and invasion assays revealed a substantial decrease in the number of migratory cells in all three cell lines following ELF3 knockdown (Figure 6h), while no significant change was observed in invasion, except in the B-CPAP cell line, where a decrease in the number of invasive cells was detected in the siELF3-transfected cells compared to the siNC-transfected condition (Figure 6i). Consequently, silencing ELF3 reproduced the functional effects of overexpressing miR-1179, with an additional effect on proliferation, making ELF3 one of the mediators of this miRNA.

### 3.6. ELF3 Silencing Decreases NOTCH3, CX3CL1, BCL2 and ALPK2 Expression

**Figure 7:**
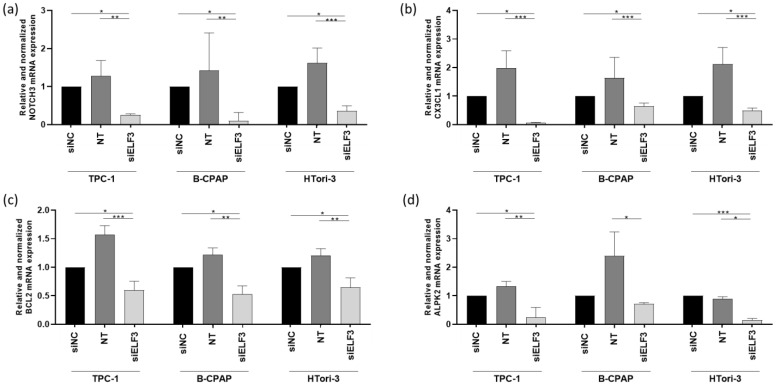
ELF3 knockdown reduces *NOTCH3*, *CX3CL1*, *BCL2* and *ALPK2* mRNA expression in TPC-1, B-CPAP and HTori-3 cells. (siNC: siRNA-negative control transfected cells, NT: non-transfected cells, siELF3: ELF3 siRNA transfected cells) (**a**) *NOTCH3*, (**b**) *CX3CL1*, (**c**) *BCL2* and (**d**) *ALPK2* mRNA expression measured by RT-qPCR four days after ELF3 siRNA transfection (TPC-1: *NOTCH3*, *n* = 7; *CX3CL1*, *n* = 8; *BCL2*, *n* = 9; *ALPK2*, *n* = 6; B-CPAP: *NOTCH3*, *n* = 11; *CX3CL1*, *n* = 9; *BCL2*, *n* = 7; *ALPK2*, *n* = 4; HTori-3: *NOTCH3*, *n* = 8; *CX3CL1*, *n* = 9; *BCL2*, *n* = 7; *ALPK2*, *n* = 10). All values relativized to their corresponding siNC (Friedman test, * *p* < 0.05, ** *p* < 0.01, *** *p* < 0.001).

Since ELF3 was the only predicted target of miR-1179 according to the miRDB and TargetScan databases, we sought to determine among the six other genes identified as downregulated by miR-1179 (Figure 4b), which ones could be regulated by ELF3. These could indeed be indirect targets of miR-1179. Their mRNA levels were quantified by RT-qPCR four days after siELF3 transfection in the three thyroid cell lines. *NOTCH3*, *CX3CL1*, *BCL2*, and *ALPK2* mRNA levels displayed consistent decrease following siELF3-mediated silencing (Figure 7), while *ITGB8* and *TGFβ-2* mRNA levels did not change.

NOTCH3 is a transmembrane receptor that is part of the Notch signaling pathway, which regulates cell fate determination [41,42]. CX3CL1 (also called fractalkine) is a chemokine existing in a membrane-bound and a soluble form, involved in immune cell recruitment and microenvironment interactions [43]. BCL2 is an anti-apoptotic protein involved in early stages of apoptosis, and ALPK2 is a serine/threonine kinase involved in the DNA damage response and maintenance of epithelial integrity [44,45,46]. All four represent interesting candidates as they could potentially be involved in tumor development. They would function as downstream effectors within a regulatory axis in which miR-1179 directly regulates the transcription factor ELF3, which itself potentially controls the transcription of *NOTCH3*, *CX3CL1*, *BCL2*, and *ALPK2*, directly or indirectly.

**Figure 8:**
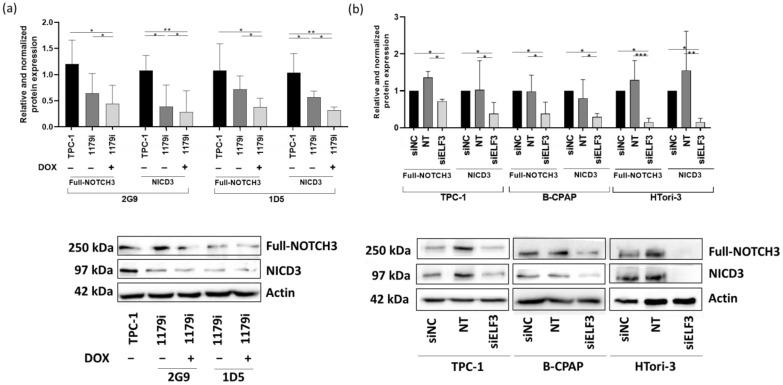
miR-1179 overexpression and ELF3 knockdown reduce NOTCH3 protein expression. Western blot analysis of full-length NOTCH3 and its active intracellular domain NICD3, normalized to actin (**a**) after miR-1179 induction in the TPC-1–1179i cell lines (2G9, 1D5) treated with doxycycline 100 ng/mL, with expression values relativized to TPC-1 cells treated with doxycycline 100 ng/mL (*n* = 7) (RM one-way ANOVA, * *p* < 0.05, ** *p* < 0.01) and (**b**) after siELF3 transfection in TPC-1, B-CPAP and HTori-3 cells, with expression values relativized to siNC (siNC: siRNA-negative control transfected cells, NT: non-transfected cells, siELF3: ELF3 siRNA transfected cells) (TPC-1: *n* = 5; B-CPAP: *n* = 6; HTori-3: *n* = 9). One representative blot is shown for each type of experiment (Friedman test, * *p* < 0.05, ** *p* < 0.01, *** *p* < 0.001).

We then decided to focus on NOTCH3 and CX3CL1, which seemed more relevant as their role in thyroid physiology is poorly documented. To confirm that their corresponding protein levels were decreased as well following miR-1179 overexpression or ELF3 knockdown, Western blot experiments were performed. We first analyzed NOTCH3 protein levels. Upon ligand binding, NOTCH3 (250 kDa) undergoes proteolytic cleavage, which generates the NOTCH3 intracellular domain (NICD3), a 97 kDa fragment able to translocate into the nucleus, where it functions as a transcriptional regulator [41,42]. Full-length NOTCH3 and NICD3 protein levels were significantly reduced following miR-1179 induction in the TPC-1-1179i+ cell lines. These decreases were already observed to some extent without doxycycline induction (Figure 8a), suggesting that the low increase in miR-1179 resulting from the leak of the expression cassette was already sufficient to downregulate NOTCH3. These results were also consistent with the decrease in ELF3 protein expression already present in the absence of doxycycline treatment (Figure 5b). We also assessed NOTCH3 protein expression following transient miR-1179 transfection in TPC-1, B-CPAP, and HTori-3 cells. In all three cell lines, both full-length NOTCH3 and NICD3 protein levels were downregulated upon miR-1179 overexpression compared to the control conditions (Supplementary Figure S7). Finally, we analyzed NOTCH3 protein expression following siELF3 transfection. Full-length NOTCH3 and NICD3 protein levels were decreased in siELF3-transfected cells compared with siNC and non-transfected controls in the three cell lines (Figure 8b). These results further support the existence of a relationship between the expression levels of miR-1179, ELF3, and NOTCH3.

Unfortunately, we were unable to investigate CX3CL1 protein levels due to a lack of reliable antibodies. Either they did not detect any protein, or they revealed bands whose molecular weights did not correspond to the expected sizes, which prevented us from drawing conclusions about protein expression.

### 3.7. ELF3, NOTCH3, and CX3CL1 Are Overexpressed in Human PTC

**Figure 9:**
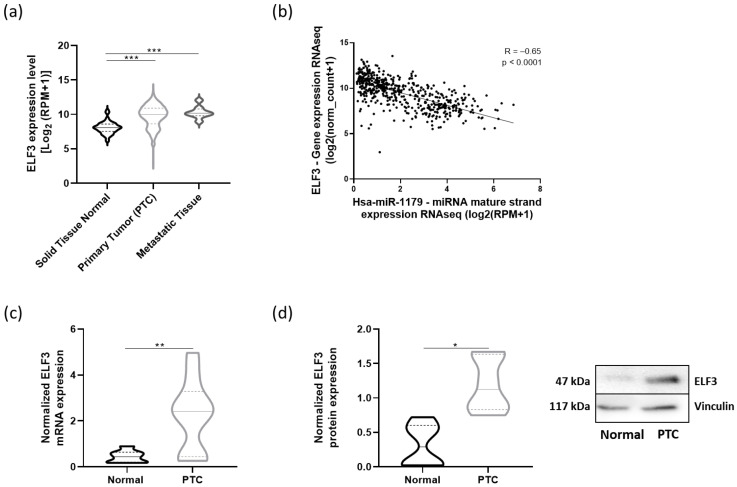
ELF3 expression is increased in human papillary thyroid cancer, and *ELF3* mRNA levels are negatively correlated with miR-1179 expression levels. (**a**) RNA-seq analysis of *ELF3* mRNA expression from The Cancer Genome Atlas (TCGA) in normal thyroids (*n* = 59), primary papillary thyroid cancers (PTC, *n* = 502), and metastatic tissues (*n* = 8) (Kruskal–Wallis test, *** *p* < 0.001). (**b**) Correlation analysis between *ELF3* mRNA and miR-1179 expressions in TCGA thyroid cancer samples (Pearson correlation, R). (**c**) RT-qPCR analysis of *ELF3* mRNA expression in independent PTC and adjacent normal thyroid tissues (*n* = 8) (Wilcoxon signed-rank test, ** *p* < 0.01). (**d**) Western blotting analysis of ELF3 protein expression normalized to vinculin expression, in PTC and adjacent normal thyroid tissues (*n* = 8). One representative blot is shown (Wilcoxon signed-rank test, * *p* < 0.05).

To address the relevance of our data in human PTC, we performed in silico analyses using the thyroid carcinoma dataset from TCGA (THCA cohort). We first investigated ELF3, i.e., the direct target of miR-1179. *ELF3* mRNA expression was significantly upregulated in both primary tumors and metastatic samples compared to normal tissues (Figure 9a) and was negatively correlated with miR-1179 expression (R= −0.65, *p* < 0.0001) (Figure 9b). The increase in *ELF3* mRNA levels was further validated by RT-qPCR in eight independent PTC samples compared with their associated non-tumoral adjacent tissues (Figure 9c). Concurrently, ELF3 protein levels were increased in PTC as well (Figure 9d). Our previous observations in cell lines were thus recapitulated in human PTC.

**Figure 10:**
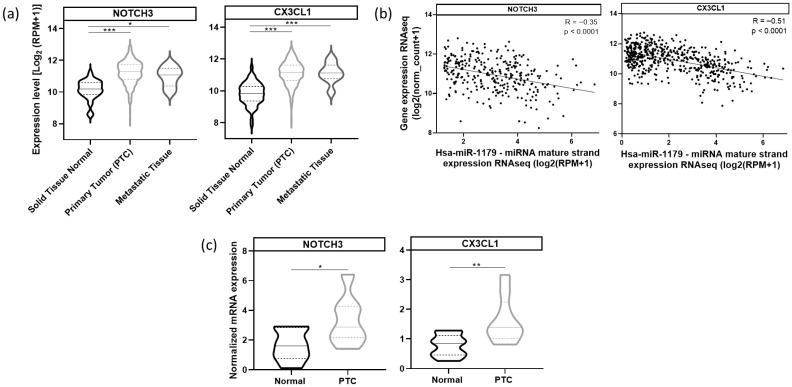
*NOTCH3* and *CX3CL1* mRNA levels are increased in human papillary thyroid cancer and are negatively correlated with miR-1179 expression levels. (**a**) RNA-seq analysis of *NOTCH3* and *CX3CL1* mRNA expression from The Cancer Genome Atlas (TCGA) in normal thyroids (*n* = 59), primary papillary thyroid cancers (PTC, *n* = 502), and metastatic tissues (*n* = 8) (Kruskal–Wallis test, * *p* < 0.05, *** *p* < 0.001). (**b**) Correlation analysis between *NOTCH3* or *CX3CL1* mRNA and miR-1179 expressions in TCGA thyroid cancer samples (Pearson correlation, R). (**c**) RT-qPCR analysis of *NOTCH3* and *CX3CL1* mRNA expression in independent PTC and adjacent normal thyroid tissues (*NOTCH3*, *n* = 7; *CX3CL1*, *n* = 9) (Wilcoxon signed-rank test, * *p* < 0.05, ** *p* < 0.01).

Next, we analyzed *NOTCH3* and *CX3CL1* mRNA expressions in the same TCGA cohort. Both *NOTCH3* and *CX3CL1* mRNAs were significantly upregulated in primary and metastatic tumor samples compared to normal tissues (Figure 10a). miR-1179 expression was negatively correlated with *NOTCH3* (R= −0.35, *p* < 0.0001) and *CX3CL1* mRNA expression (R= −0.51, *p* < 0.0001) (Figure 10b). These findings were corroborated by RT-qPCR analyses demonstrating increased *NOTCH3* and *CX3CL1* mRNA expression in independent PTC compared to adjacent normal tissues (Figure 10c). These data suggest the existence, in the human thyroid, of a pathway involving miR-1179, which targets ELF3, itself a potential regulator of NOTCH3 and CX3CL1 expression, and whose alteration in PTC may contribute to tumor development.

## 4. Discussion

Thyroid cancer represents the most common malignancy of the endocrine system and has shown a steadily increasing incidence over recent decades [47]. Although papillary thyroid carcinoma generally has a favorable prognosis, a subset of tumors develop recurrence or undergo dedifferentiation, so that a better understanding of the molecular mechanisms involved in this pathology could lead to new treatment options [5].

MicroRNAs (miRNAs) have emerged as important regulators of gene expression and represent valuable tools for cancer characterization due to their stability, reliable detection, and capacity to modulate multiple targets. In the present study, we sought to elucidate the role of miR-1179 in papillary thyroid carcinoma (PTC) tumorigenesis. This miRNA was selected based on previous miRNA profiling performed in our laboratory, which identified miR-1179 as one of the most significantly downregulated miRNAs in PTC [18]. Notably, miR-1179 expression was further decreased in lymph node metastases and in tumors harboring the BRAF^V600E^ mutation, suggesting an association between miR-1179 loss and increased tumor aggressiveness or risk of recurrence [18,48]. Furthermore, there is evidence linking miR-1179 to thyroid function. Grasberger et al. reported that inherited mutations in a short tandem repeat (STR) activate a primate-specific enhancer that upregulates *MIR7-2/MIR-1179*, leading to thyrotropin resistance [19]. Another study reported the identification of a single-nucleotide polymorphism near the *MIR1179* locus associated with variation in TSH levels [21]. In both cases, the biological mechanisms involved are unknown, and the functional role of miR-1179 in normal thyroid physiology and cancer development remains largely unexplored. This study aimed to determine the function of miR-1179 in thyroid cells and to identify the molecular pathways targeted by this miRNA.

We first verified the expression of miR-1179 in PTC using the miRNA sequencing data from the thyroid carcinoma (THCA) cohort of The Cancer Genome Atlas (TCGA), together with analyses performed on independent papillary thyroid cancers and their adjacent normal thyroid tissues. Collectively, these data confirmed the pronounced downregulation of miR-1179 in PTC, in line with our previous and other reports [18,48,49]. They support the hypothesis that the strong downregulation of miR-1179 may contribute to tumor progression.

Based on these human PTC-derived observations, we next sought to investigate the functional consequences of miR-1179 downregulation using in vitro models. Consistent with the PTC data, miR-1179 expression was uniformly reduced across the panel of thyroid cell lines employed in this study, two cancer-derived cell lines covering the main oncogenic alterations present in PTC (TPC-1, B-CPAP), and the non-tumorigenic HTori-3 cell line, thereby providing biologically relevant models to assess the impact of miR-1179 restoration in a tumoral and non-tumoral context. These models were subsequently used to perform functional assays and to explore the molecular pathways modulated by miR-1179 overexpression.

To further investigate the upstream molecular mechanisms that might contribute to miR-1179 downregulation in thyroid cancer, we examined miR-1179 expression in the TPC-1 and B-CPAP cell lines following treatment with 5′-aza-2′-deoxycytidine, a demethylating agent, or SAHA, a histone deacetylase inhibitor. Both treatments restored miR-1179 expression, suggesting that epigenetic alterations may, at least in part, contribute to its downregulation in thyroid cancer. In line with our data, DNA methylation of the miR-1179-coding gene has been described in non-small-cell lung cancer [50]. To our knowledge, no studies have specifically investigated the epigenetic regulation of miR-1179 in thyroid cancer. However, similar mechanisms have been described for other microRNAs, such as miR-204-5p [38], supporting the idea that epigenetic dysregulation may represent a broader mechanism contributing to microRNA downregulation in this cancer type.

To model miR-1179 re-expression in a more controlled manner, we generated two doxycycline-inducible miR-1179-expressing cell lines derived from wild-type TPC-1 cells (TPC-1-1179i cells: 2G9 and 1D5). A low level of basal miR-1179 expression was detected in the absence of doxycycline, likely due to promoter leakage from the doxycycline-dependent promoter *P_TRE3G_*, but this leakiness remained low compared with the robust induction achieved upon doxycycline treatment (TPC-1-1179i+ cells) (Figure S1). Unfortunately, leaks are frequently observed in inducible gene expression systems, and this is a limitation of our inducible model because it prevented us from having the corresponding controls (uninduced TPC-1-1179 cells). We viewed it as an opportunity to analyze the impact of a moderate increase in miR-1179 levels and a more pronounced increase observed following doxycycline induction, compared to WT TPC-1 cells, which were then used as controls. To further strengthen our experimental approach, transient miR-1179 transfections in TPC-1, B-CPAP, and HTori-3 cells were also performed, and only results consistent with those observed in the inducible cell lines were considered.

Functional analyses have revealed that miR-1179 inhibits cell growth, a feature that can be attributed to increased apoptotic activity, as demonstrated in both inducible and transiently transfected cell lines. They are consistent with previous reports implicating miR-1179 in apoptosis-related processes in thyroid cancer, notably through its interaction with circ_0039411 and circ_0101622 [30,31], as well as in cervical cancer through an interaction with circ_0000263 [51]. Those three circular RNAs are able to bind and sponge miR-1179. In contrast, no effect of miR-1179 overexpression was observed on EdU incorporation into DNA during the S phase of the cell cycle, suggesting that it has no impact on cell cycle progression in our experimental models. This observation differs from previous reports describing a potential role for miR-1179 in cell proliferation [24,25,26,50,51,52,53]. Such discrepancies could be related to differences in the genetic background of the cell lines used, as this effect has not been consistently observed in thyroid cell lines. Additionally, several of these studies have assessed proliferation using metabolic assays such as MTT, MTS, or CCK-8, which primarily reflect cell viability at the population level rather than direct DNA synthesis. Taken together, these results suggest that the primary growth-inhibitory effect of miR-1179 in thyroid cells is mediated through apoptosis rather than direct modulation of cell cycle progression. To complete the functional characterization, we further assessed the migratory and invasive capacities of our thyroid cell lines. In both our inducible and transient models, the overexpression of miR-1179 led to a marked reduction in cell migration, whereas no significant effect was observed on their invasive potential in our experimental conditions. Although several studies have reported an association between miR-1179 and migratory behavior in various other, non-thyroid, cell types, together with a role in invasion, we did not observe such a role in the thyroid cell lines [24,26,54]. The difference may be attributable to cell-specific transcriptomic contexts or to differences in the molecular programs regulating migration and invasion in thyroid cells. While migration and invasion are distinct processes, migratory capacity is required for invasive behavior, and our data so far support a role for miR-1179 in migration [55].

It is important to note that these functional effects (increased apoptosis, inhibition of cell migration) were observed only at high, supraphysiological levels of miR-1179, achieved following doxycycline induction or transient transfection of miR-1179. To minimize potential off-target effects while maintaining efficient modulation of the target, we used a concentration of 5 nM for the transfection experiments, unlike most published studies, which used concentrations ranging from 20 to 200 nM. Only the negative effect of miR-1179 on cell viability was already observed in the absence of doxycycline, i.e., at a lower concentration. The limited effects observed following a small increase in miRNA levels likely reflect the fact that miRNAs function as a network and that the dysregulation of a single miRNA is generally not sufficient to disturb the cell homeostasis. miRNAs have moderate effects that serve to fine-tune regulation, and since they target many genes, it is likely that a compensatory mechanism will develop. In the context of thyroid cancer, we have previously shown that the decreased expression of miR-7-5p and miR-204-5p may contribute to thyroid tumorigenesis. MiR-7-5p inhibits proliferation by targeting the EGFR/MAPK and IRS2/PI3K signaling pathways, while miR-204-5p inhibits cell invasion by regulating several targets associated with EMT, such as HMGA2 [38,56]. It is therefore conceivable that the combined consequences of reduced expression of these three miRNAs, along with other dysregulations, may promote thyroid tumor development.

It is well established that miRNAs regulate gene expression through binding to the 3′ untranslated regions (3′UTRs) of their target mRNAs, leading to mRNA degradation and/or translational repression [16]. So far, very few mRNA targets of miR-1179 have been identified. To gain further insight into the molecular mechanisms underlying the effects of miR-1179 in thyroid cells, we performed RNA sequencing analysis on cells overexpressing miR-1179. An exploratory analysis (FDR < 0.1) identified a subset of differentially expressed genes, common to both our inducible and transient models, that represent potential mRNA targets of miR-1179. We subsequently validated the downregulation of *ELF3*, *NOTCH3*, *CX3CL1*, *ALPK2*, *ITGB8*, *BCL2*, and *TGFβ-2* mRNA in the inducible TPC-1 cell lines. Although the system demonstrated a strong inducible response, we cannot fully exclude the possibility of functional consequences resulting from minimal residual activity due to leakage of expression, as indicated by modest changes in a subset of downstream targets under non-induced conditions. When performing those analyses in the transiently transfected cell lines, we noticed that the miRNA mimic negative control (Cel-miR-67, MIMAT0000039) unexpectedly impacted the expression of several investigated mRNAs. The mature Cel-miR-67 sequence has minimal sequence identity with human miRNAs; however, in silico analysis of miRDB (https://mirdb.org/, accessed on 13 April 2026) for miRNA target prediction identified more than 300 potential binding sites for Cel-miR-67 in the human transcriptome. Although this result was not anticipated, it is consistent with the well-established capacity of miRNAs to bind their target transcripts through a short seed sequence (6–8 nucleotides), which may occur frequently across the transcriptome, thereby increasing off-target interactions. This provides a plausible explanation for the transcriptional changes observed in our RT-qPCR experiments and highlights an important limitation of this commonly used negative control. To overcome this drawback, we included miR-204-5p as a control to assess the specificity of the effects of miR-1179. miR-204-5p had no or only a minor impact on the expression of the mRNAs analyzed and was therefore a more appropriate control for most of these genes. Its biological activity was demonstrated by the downregulation of its established mRNA target *BCL2*, as previously reported [39]. Together with the data obtained with the inducible cell lines, these findings convinced us that the effects of miR-1179 were indeed specific. It should be noted that the non-specific effects of Cel-miR-67 were observed in the RT-qPCR experiments, but not in the functional assays. So, although Cel-miR-67 is commonly used as a negative control in our laboratory and others, our data indicate that it should be used with caution. The use of inducible cell lines avoids this problem but can lead to other issues related to negative control, such as leakage from the expression cassette, as this study demonstrates. This underscores the importance of obtaining consistent results using different experimental models.

Several of the downregulated genes identified by RNA sequencing and subsequently validated are known modulators of apoptotic pathways, such as *ELF3*, *NOTCH3*, *CX3CL1*, *ALPK2*, and *BCL2*. Those genes have been described as promoting cell survival, and their downregulation upon miR-1179 overexpression is consistent with the increased apoptotic phenotype observed [40,45,46,57]. Regarding the observed effects on cell migration, ELF3, CX3CL1, ALPK2, and TGFβ-2 have been reported to promote migratory and/or invasive behaviors in various cellular contexts. Their concomitant downregulation in miR-1179-expressing thyroid cells may therefore contribute to the reduced migratory capacity observed in our models [40,58,59].

By intersecting our RNA-seq dataset with two independent miRNA target prediction databases, we identified ELF3 as the sole potential target of miR-1179. ELF3 is a member of the ETS family of transcription factors, mainly expressed in epithelial cells, and its dysregulation has been implicated in the progression of various cancers. In this context, ELF3 emerged as a particularly compelling target, given its ability to regulate the expression of numerous downstream genes and to influence multiple cellular pathways [40]. Given *ELF3* mRNA downregulation upon miR-1179 expression, we further evaluated ELF3 protein expression and observed a corresponding decrease. To validate that ELF3 is a direct downstream target of miR-1179, we performed a luciferase reporter assay, providing evidence that miR-1179 directly binds to the predicted miR-1179 binding site located in the 3′UTR of *ELF3*.

To investigate the role of ELF3 in the context of the biological functions of miR-1179 in thyroid cells, functional analyses with ELF3 siRNA were carried out and revealed that ELF3 knockdown inhibited cell growth, induced apoptosis, and inhibited migration, thereby reproducing the functional effects of miR-1179. However, ELF3 knockdown additionally inhibited EdU incorporation into DNA in TPC-1 and B-CPAP cells. This supplementary effect may be explained by the stronger and more direct inhibition of ELF3 achieved with siRNA compared to miR-1179-mediated downregulation (compare Figure 5b and Figure 6b). Furthermore, although ELF3 is one mediator of the effects of miR-1179, the latter exerts broader regulatory effects, as miRNAs target multiple transcripts and act as fine-tuners, which could attenuate the specific contribution of ELF3 repression. In accordance with our results, other studies have also reported a role for ELF3 in regulating cell proliferation [60,61,62]. While the negative effects of miR-1179 on cell growth appear to be mainly driven by apoptosis induction, ELF3 inhibition likely reflects a combined effect of increased apoptosis and reduced cell proliferation, which is consistent with its stronger effect on cell growth (Figure 2a and Figure 6c). No significant effects were observed on cell invasion following ELF3 inhibition, except for B-CPAP cells, in which invasion was significantly reduced. Together, these results suggest that ELF3 acts primarily as an anti-apoptotic factor and a promoter of cell proliferation and migration in the thyroid cellular context. Overall, these findings agree with the study by Chen et al., who reported that silencing ELF3 in *BRAF*-mutated thyroid cancer cell lines, including the B-CPAP cells, significantly reduced cell growth, migration, and invasion [62]. The divergent responses to invasion observed between the B-CPAP cells and the two other cell lines could be related to the more aggressive nature of the B-CPAP cells, derived from a poorly differentiated thyroid carcinoma carrying the BRAF^V600E^ mutation, but this remains to be explored. Our results are also in line with previous studies in other tumor types, including ovarian cancer, non-small-cell lung cancer, and hepatocellular carcinoma, in which ELF3 has been shown to promote EMT, thereby enhancing both migration and invasion [40,61,63], although in our thyroid cell models, the predominant effect was observed on migration rather than invasion. To further clarify the role of ELF3, rescue experiments should be performed by reintroducing an ELF3 construct lacking the miR-1179 binding site in the 3′ UTR, while maintaining miR-1179 overexpression. If the phenotypic effects induced by miR-1179 are then reversed, this would support the conclusion that ELF3 is an important mediator of miR-1179 activity.

Taken together, these observations suggest that ELF3 may exert conserved pro-tumorigenic functions in various tissue contexts, including thyroid cancer, and highlight the need for further studies to better understand its broader regulatory role. Through its DNA-binding activity, ELF3 regulates a wide spectrum of genes and can thereby promote oncogenic processes. It enhances β-catenin transactivation in colorectal cancer (135), its expression correlates with EMT processes, and it regulates proliferation and apoptosis via FOXM1 and NOTCH3 [40,64,65]. However, despite these described roles in other malignancies, the downstream target landscape of ELF3 in thyroid cells remains poorly characterized.

ELF3 was the only gene whose expression was downregulated by miR-1179, and that contained a predicted binding site for miR-1179, prompting us to investigate whether the additional downregulated genes could be linked to the miR-1179-ELF3 regulatory axis, i.e., indirectly regulated by miR-1179 via *ELF3*. *NOTCH3*, *CX3CL1*, *BCL2*, and *ALPK2* mRNA were significantly downregulated upon ELF3 silencing, supporting their potential role as downstream direct or indirect targets of ELF3. This last point is something that should be clarified in future studies.

NOTCH3 is a transmembrane receptor of the Notch family, and ELF3 binding sites have been identified in the proximal promoter region of the NOTCH3 gene [65,66], suggesting that NOTCH3 may be activated directly at the transcriptional level by ELF3. However, this has yet to be confirmed experimentally in our cellular models. NOTCH3 is activated by binding to a ligand present on the cell surface of a neighboring cell. Upon ligand binding, NOTCH3 undergoes proteolytic cleavage to release the NOTCH3 intracellular domain (NICD3), which translocates to the nucleus. There, NICD3 functions as a transcriptional coactivator by binding to CSL, displacing transcriptional repressors, and recruiting coactivators to induce the expression of Notch target genes. The Notch signaling pathway regulates cell differentiation, proliferation, and survival, with oncogenic or tumor-suppressive roles depending on the cell type, although it acts primarily as an oncogene [41,42,67]. In accordance with our data, an ELF3-NOTCH3 signaling axis has been described in lung and pancreatic adenocarcinoma [40,65,68], highlighting a potentially conserved regulatory pathway. CX3CL1 is a chemokine that regulates cell migration through two mechanisms: as a transmembrane protein, it mediates cell adhesion, and following cleavage by metalloproteases, it is released as a soluble factor that recruits immune cells via a chemoattractant gradient [69]. While no studies have directly linked miR-1179 to immune modulation in thyroid cancer, previous work in pancreatic carcinoma identified miR-1179 as part of an immune-related prognosis [70]. Our data suggests a potential role for miR-1179, ELF3, and CX3CL1 in shaping the immune landscape of thyroid tumors. BCL2 (B-Cell Lymphoma 2) is an anti-apoptotic protein that regulates cell survival by inhibiting programmed cell death [45,46]. Finally, ALPK2 is a member of the alpha-kinase family, involved in cell cycle regulation, apoptosis, and EMT [44,57,59], and whose regulation by ELF3 has not been previously reported. We further explored the regulation of NOTCH3 and CX3CL1, as data on their role in thyroid cells are scarce. We confirmed that NOTCH3 protein levels were decreased following miR-1179 overexpression or ELF3 inhibition, but were unable to study CX3CL1 protein levels due to the lack of reliable antibodies available.

To further elucidate the downstream molecular consequences of ELF3 loss in thyroid cells, RNA sequencing following ELF3 silencing could provide valuable insight into ELF3-dependent transcriptional programs. Alternatively, chromatin immunoprecipitation sequencing (ChIP-seq) would allow the identification of ELF3 DNA-binding sites and, in fine, its direct downstream effectors. This would help to establish whether ELF3 directly activates *NOTCH3*, *CX3CL1*, *BCL2*, and *ALPK2* at the transcriptional level.

Although in vitro cell culture models are indispensable experimental tools, their ability to accurately reflect the physiological complexity of in vivo biological systems remains limited, notably due to the absence of a native tissue microenvironment, phenotypic drift, and the oversimplification of cellular interactions [71,72,73]. Several alternative approaches can be considered to deepen our understanding of the role of miR-1179 and its downstream targets in thyroid cancer. First, the use of thyroid organoids will provide a more physiologically relevant system, enabling advanced investigation of miR-1179 and ELF3 roles within a three-dimensional, tissue-like context [74,75]. Second, in vivo studies in mice could be valuable; however, as miR-1179 is primate-specific, such models would only allow the study of ELF3 function in thyroid tumorigenesis. Various mouse models of thyroid cancer are available to address this issue.

To evaluate the relevance of our findings for human PTC, the expression levels of miR-1179, *ELF3*, *NOTCH3*, and *CX3CL1* were investigated in this cancer. We performed in silico analyses using the THCA cohort from TCGA, complemented by in vivo analyses performed on independent human PTC. Collectively, these data demonstrated a marked upregulation of *ELF3*, *NOTCH3*, and *CX3CL1* in PTC. Accordingly, both ELF3 and CX3CL1 have been reported to be upregulated in PTC by other groups [62,74,75]. These findings suggest that loss of miR-1179 may contribute to tumor progression by promoting the expression of ELF3, NOTCH3, and CX3CL1, thereby complementing the activation of the MAPK and PI3K signaling pathways. These proteins represent new potential therapeutic targets that warrant further mechanistic and translational validation.

Our observations are based on data obtained from thyroid cancer-derived cell lines but also from a non-tumorigenic cell line (HTori-3), suggesting that miR-1179 appears to play an essential role in thyroid physiology. While its downregulation may contribute to the development of PTC, its upregulation could play a role in inherited thyrotropin resistance, a genetic disease characterized by molecular defects that hinder the transmission of the TSH signal into thyrocytes. In this pathology, STR mutations activate a thyroid-specific enhancer of *MIR7-2/MIR1179*, leading to increased expression of both miRNA [19]. The pro-apoptotic effect of miR-1179 overexpression identified in this study, combined with the well-established antiproliferative effect of miR-7-5p overexpression, could contribute to the reduced sensitivity of the thyrocytes to the stimulatory action of TSH on function and proliferation [56]. Further studies should help to better define their respective roles in the pathophysiology of thyrotropin resistance.

## 5. Conclusions

This work provides new insight into the molecular mechanisms underlying papillary thyroid cancer development. Our data show that miR-1179 exerts an inhibitory effect on thyroid cell growth through its pro-apoptotic activity, as well as on cell migration, suggesting that its downregulation may contribute to the development of PTC. We identified and experimentally validated a novel direct target of miR-1179, ELF3, as well as additional targets, including NOTCH3 and CX3CL1, both indirectly regulated by miR-1179 via ELF3. We further demonstrated that miR-1179 effects on cell apoptosis and migration are mediated by ELF3. Finally, we revealed the existence of an inverse correlation between the decreased expression of miR-1179 and the increased expression of *ELF3*, *NOTCH3*, and CX3CL1 mRNA in human PTC. These findings suggest that loss of miR-1179 may contribute to tumor progression by promoting the expression of *ELF3*, *NOTCH3*, and *CX3CL1*, highlighting new potential therapeutic perspectives for the treatment of thyroid cancer.

## Data Availability

The original contributions presented in the study are included in the article/Supplementary Materials; further inquiries can be directed to the corresponding author.

## Supplementary Materials

The following supporting information can be downloaded at: https://www.mdpi.com/article/10.3390/cells15090802/s1, Table S1: Clinicopathological features of PTC patients. Table S2: Primer sequences list. Supplementary Figure S1: Overexpression of miR-1179 in our different experimental models. Supplementary Figure S2: Doxycycline treatment of TPC-1 cells does not modify miR-1179 expression and has no functional effects. Supplementary Figure S3: Effects of miR-1179 overexpression in transiently transfected TPC-1, B-CPAP and HTori-3 cells. Supplementary Figure S4: RT-qPCR analysis of miR-1179 downregulated genes in transfected TPC-1, BCPAP and HTori-3 cells. Supplementary Figure S5: Western blot analysis of ELF3 following miR-1179 transfection in TPC-1, B-CPAP, and HTori-3 cells. Supplementary Figure S6: Transient siELF3 transfection in TPC-1 cells shows effective knockdown of ELF3 at both mRNA and protein levels. Supplementary Figure S7: Western blot analysis of NOTCH3 following miR-1179 transfection in TPC-1, B-CPAP, and HTori-3 cells.

## Abbreviations

The following abbreviations are used in this manuscript:

ALPK2	Alpha Kinase 2
BAX	BCL2-Associated X Protein
BCL2	B-Cell Lymphoma 2
CSL	CBF1/Su(H)/LAG-1 family transcription factor
CX3CL1	C-X3-C Motif Chemokine Ligand 1
EdU	5-Ethynyl-2′-Deoxyuridine
ELF3	E74 Like ETS Transcription Factor 3
FDR	False Discovery Rate
ITGB8	Integrin Subunit Beta 8
NICD3	NOTCH3 Intracellular Domain
NOTCH3	Notch Receptor 3
NT	Non-Transfected
NC	Negative Control
PTC	Papillary Thyroid Carcinoma
TCGA	The Cancer Genome Atlas
THCA	Thyroid Carcinoma (TCGA cohort)
TGFβ-2	Transforming Growth Factor Beta 2
TSH	Thyroid-Stimulating Hormone
