# Supplementary material for: New Insights into the Development of Papillary Thyroid Cancer: The Roles of miR-1179 and ELF3
Source: Cells. 2026 Apr 29;15(9):802. doi: 10.3390/cells15090802 (PMC13163138; doi:10.3390/cells15090802)
Supplement: Supplementary file 1 [file cells-15-00802-s001.zip › cells-4250239-supplementary.pdf]

Table S1. Clinicopathological features of PTC patients. All patients were tested BRAF^{V600E} negative, except patient 2.

Patient ID	Diagnosis	Gender	Age	TNM
1	PTC, classic	F	45	pT1bN1a
2	PTC, classic	F	76	pT1bNx
3	PTC, follicular	M	61	pT1bNx
4	variant	F	35	pT1b(m)N0
5	PTC, classic	F	28	pT3N1b
6	PTC, classic	F	32	pT1N1b
7	PTC, classic	F	30	pT1b(m)
8	PTC, classic	F	51	pT1N0
9	PTC, classic	F	68	pT3N0
10	PTC, classic	F	23	pT2N0
11	PTC, follicular variant	M	14	pT3(M)N1b
	PTC, diffuse sclerosing variant			

Table S2. Primer sequences list. All sequences should be read 5′ to 3′.

Human Gene ID	Primer Forward	Primer Reverse
ELF3	CAACTATGGGGCCAAAAGAA	TTCCGACTCTGGAGAACC
NOTCH3	GCTCATCGCCAGCCATGC	TTGAGCAGGGCCAAAGTG
CX3CL1	CCTGTAGCTTTGCTCATCCA	CCTTGACCCATTGCTCCTT
ALPK2	AATGCCCAAACTCGAAACATC	GGCAGCTTAATTTTACATTTCCA
ITGB8	TTGTATGCAATGCCTTCACC	CTCAAGTAGCTTGGGCTGGA
BCL2	GATTGTGGCCTTCTTTGA	CAAACTGAGCAGAGTCTTC
BAX	GATGCGTCCACCAAGAAG	AGTTGAAGTTGCCGTCAG
TGFβ-2	TGCTGCACTTTTGTACCATCT	GGGGTCTTCCCACTGTTTTT
NEDD8	TGACCGGAAAGGAGATTGAGAT	CCTCCACACGCTCCTTGATT
TTC1	CGGAGAAGCTGTGAGGTTCTTA	TCCTCTGGAACCCCACAGTT

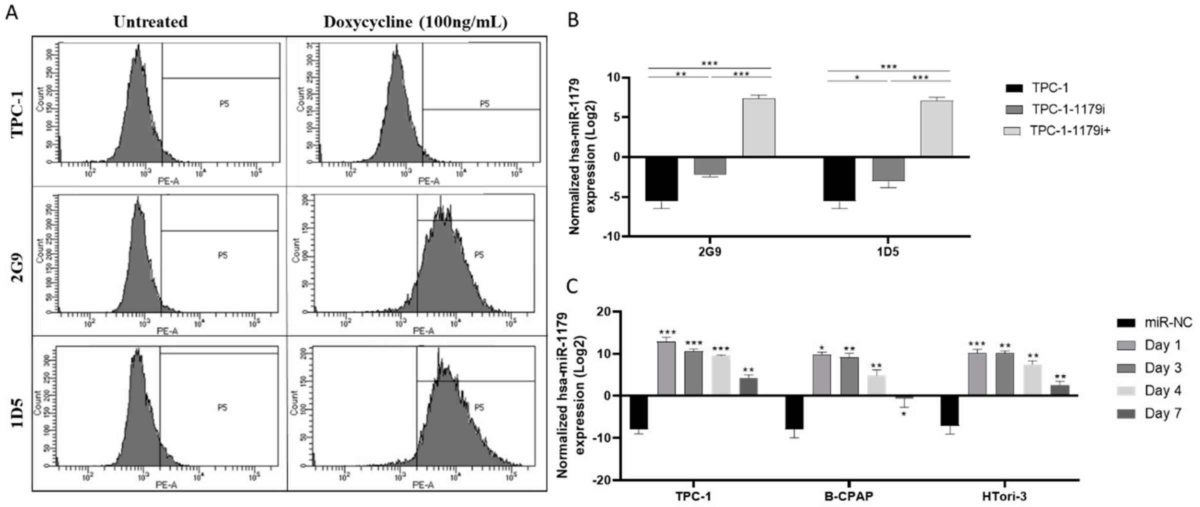

Supplementary Figure S1. Overexpression of miR-1179 in our different experimental models. (**A, B**) miR-1179 induction following doxycycline addition (100 ng/mL) in the TPC-1-1179i cell lines (2G9, 1D5) analyzed four days after treatment (**A**) by flow cytometry by assessing TurboRFP expression (tRFP positive cells, P5) and (**B**) by RT-qPCR analysis of miR-1179 expression (*n*=7). (Friedman test, *p<0.05; **p<0.01; ***p<0.001. (**C**) RT-qPCR analysis of miR-1179 levels following miR-1179 transient transfection in the TPC-1, B-CPAP and HTori-3 cell lines, measured 1, 3, 4, and 7 days after transfection (miR-NC: negative control mimic) (*n*=3; Two-way ANOVA, *p < 0.05, **p < 0.01, ***p < 0.001 vs. miR-NC cells).

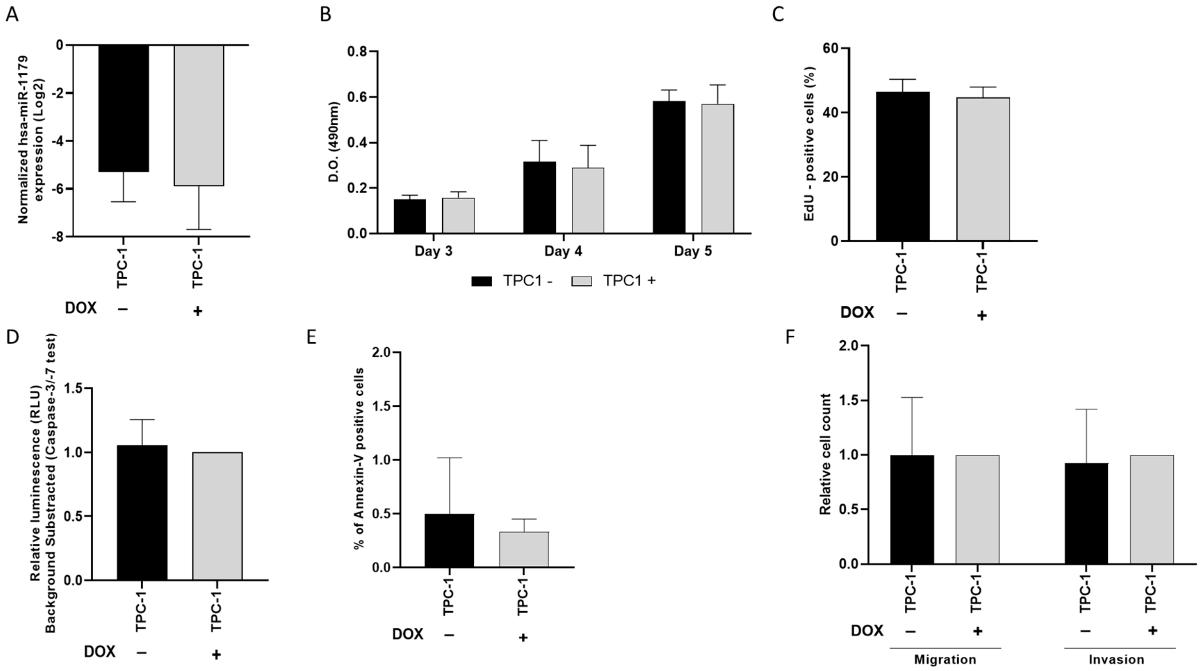

Supplementary Figure S2. Doxycycline treatment of TPC-1 cells does not modify miR-1179 expression and has no functional effects. Analyses were performed four days after exposure to 100 ng/mL doxycycline, TPC-1- indicates untreated cells, whereas TPC-1+ refers to cells treated with doxycycline. **(A)** miR-1179 expression measured by RT-qPCR (*n*=7). **(B)** Cell viability, quantified by absorbance at 490 nm 3, 4 and 5 days after treatment and presented as mean daily values for each condition (*n*=8). **(C)** Cell proliferation, evaluated by EdU incorporation and flow cytometry (*n*=4). **(D)** Caspase-3/7 activity, measured by luminescence (RLU) and normalized to background (n = 4). **(E)** Apoptosis, assessed by annexin-V staining and flow cytometry (*n*=5). **(F)** Cell migration and invasion, quantified as the normalized number of migratory and invasive cells from five random microscopic fields per condition (Transwell assay) (*n*=6).

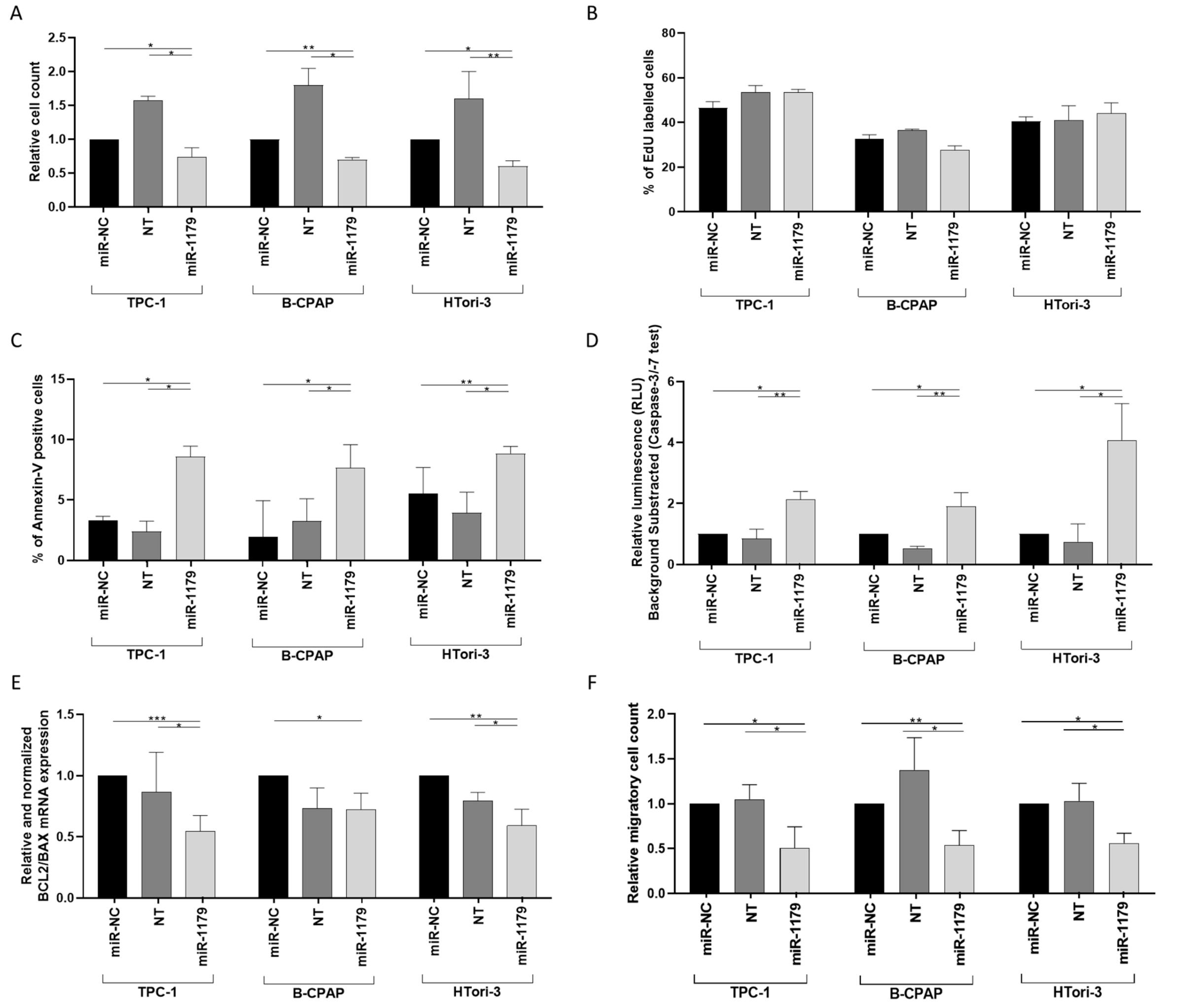

Supplementary Figure S3. Effects of miR-1179 overexpression in transiently transfected TPC-1, B-CPAP and HTori-3 cells. (miR-NC: Cel-miR-67 tranfected cells, NT: non-transfected cells, miR-1179: miR-1179 transfected cells) (**A**) Cell counting at day 3 post-transfection and relativized to miR-NC cells (*n*=7) (**B**) EdU incorporation assessed by flow cytometry and relativized to miR-NC cells (*n*=4). (**C-E**) Apoptosis assessed (**C**) 24 hour post transfection by Annexin-V staining using flow cytometry (TPC-1, *n*=5; B-CPAP, *n*=6; HTori-3, *n*=4), (**D**) 4 days post transfection by Caspase-3/-7 activity measurement by luminescence (RLU) assays, normalized to background (TPC-1, *n*=9; B-CPAP, *n*=7; HTori-3, *n*=6) and (**E**) by *BCL2* and *BAX* mRNA levels quantification by RT-qPCR, expressed as the *BCL2*/*BAX* mRNA expression ratio relativized to miR-NC cells (*n*=6) (**F**) Quantification of migratory cells from five random fields per condition and relativized to miR-NC cells (Transwell assay) (*n*=6). (Friedman test, *p<0.05, **p<0.01, ***p<0.001).

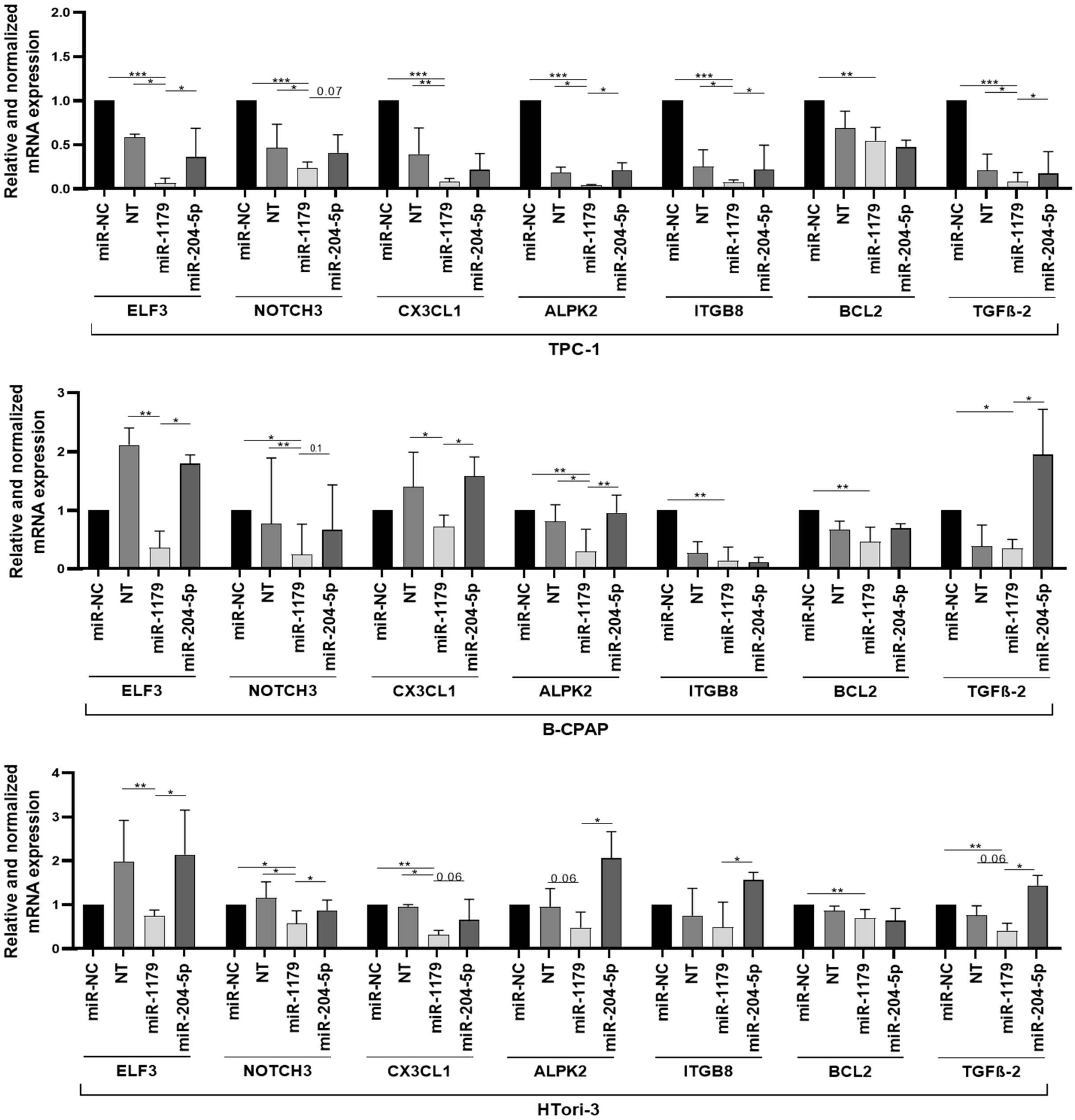

Supplementary Figure S4. RT-qPCR analysis of miR-1179 downregulated genes in transfected TPC-1, BCPAP and HTori-3 cells. (miR-NC: Cel-miR-67 transfected cells, NT: non-transfected cells, miR-1179: miR-1179 transfected cells, miR-204-5p: miR-204-5p transfected cells) mRNA expression of *ELF3*, *NOTCH3*, *CX3CL1*, *ALPK2*, *ITGB8*, *BCL2*, and *TGFB-2* quantified by RT-qPCR 4 days after transfection. Transcripts levels were relativized to miR-NC expression. Cells transfected with miR-204-5p were included as an additional negative control. Sample sizes: TPC-1: *ELF3* (*n*=11), *NOTCH3* (*n*=8), *CX3CL1* (*n*=8), *ALPK2* (*n*=10), *BCL2* (*n*=9), *TGFβ-2* (*n*=6), *ITGB8* (*n*=9); B-CPAP: *ELF3* (*n*=5), *NOTCH3* (*n*=5), *CX3CL1* (*n*=6), *ALPK2* (*n*=6), *ITGB8* (*n*=8), *BCL2* (*n*=6), *TGFβ-2* (*n*=10); HTori-3: *ELF3* (*n*=6), *NOTCH3* (*n*=6), *CX3CL1* (*n*=4), *ALPK2* (*n*=7), *ITGB8* (*n*=8), *BCL2* (*n*=6), *TGFβ-2* (*n*=8) (Friedman test, *$p < 0.05$, **$p < 0.01$, ***$p < 0.001$).

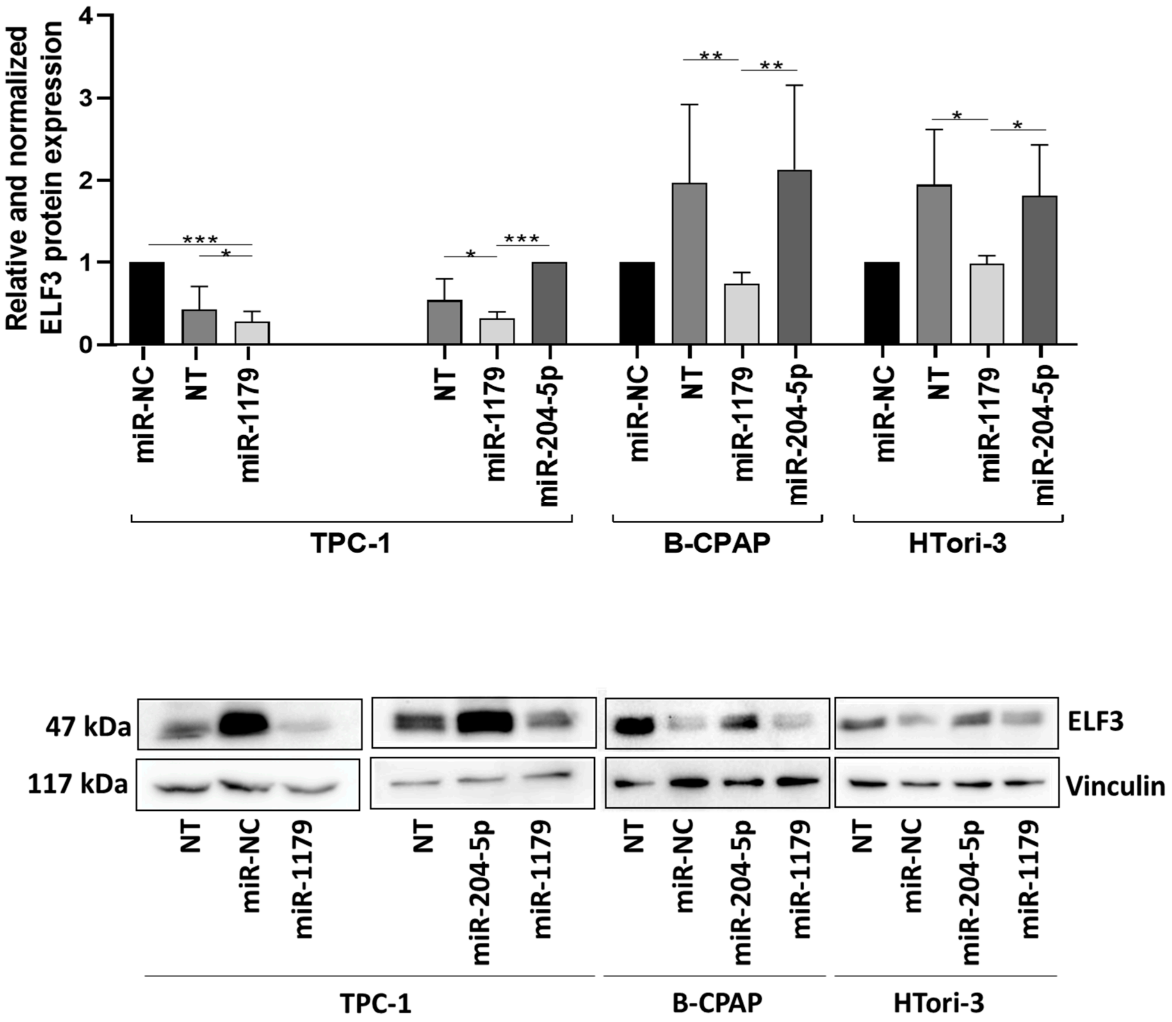

Supplementary Figure S5. Western blot analysis of ELF3 following miR-1179 transfection in TPC-1, B-CPAP, and HTori-3 cells. (miR-NC: Cel-miR-67 transfected cells, NT: non-transfected cells, miR-1179: miR-1179 transfected cells, miR-204-5p: miR-204-5p transfected cells, included as an additional negative control). Western blot analyses performed 4 days after transfection, and quantification of ELF3, normalized to vinculin and relativized to miR-NC (or miR-204-5p for TPC-1 in the absence of miR-NC, second panel). (TPC-1 (miR-NC), *n*=5; TPC-1 (miR-204-5p), *n*=6; B-CPAP, *n*=6; HTori-3, *n*=6) (Friedman test, *p<0.05, **p<0.01, ***p<0.001).

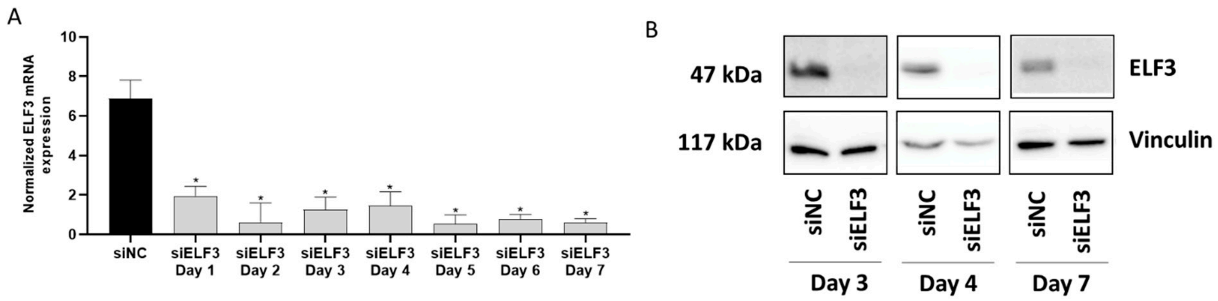

Supplementary Figure S6. Transient siELF3 transfection in TPC-1 cells shows effective knockdown of ELF3 at both mRNA and protein levels. (siNC: siRNA-negative control transfected cells, siELF3: ELF3 siRNA transfected cells) (**A**) *ELF3* mRNA expression from day 1 to day 7 quantified by RT-qPCR (n=3; RM one-way ANOVA, *$p < 0.05$). (**B**) ELF3 protein expression assessed by Western blot at days 3, 4, and 7 following siELF3 transient transfection.

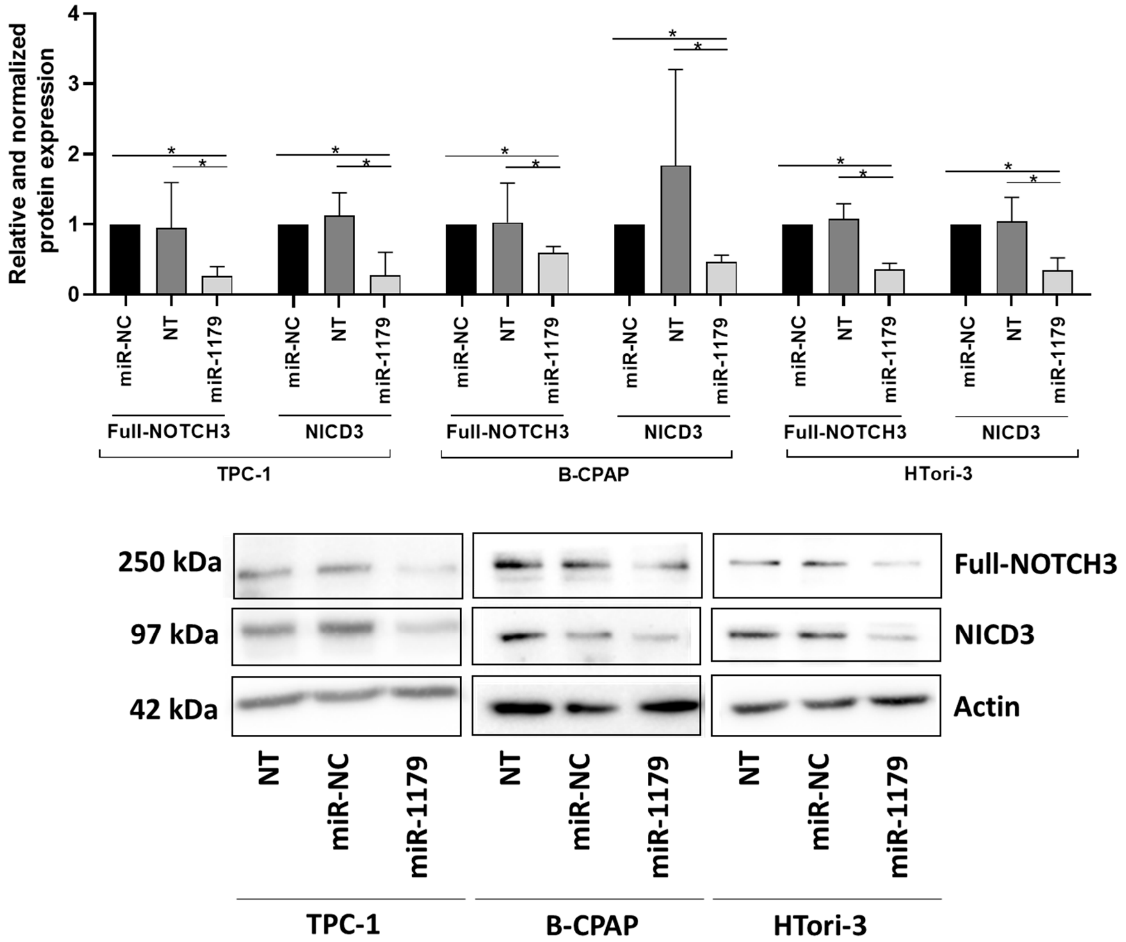

Supplementary Figure S7. Western blot analysis of NOTCH3 following miR-1179 transfection in TPC-1, B-CPAP, and HTori-3 cells. (miR-NC: Cel-miR-67 transfected cells, NT: non-transfected cells, miR-1179: miR-1179 transfected cells). Western blot analyses performed 4 days after transfection and quantification of full-length NOTCH3 and its active intracellular domain NICD3, normalized to actin and relativized to miR-NC expression (n=6) (Friedman test, *p<0.05).
